# Biocompatible chitosan-coated polydatin@ZIF-8 MOF nanostructures: synthesis, drug delivery and enhanced antibacterial properties

**DOI:** 10.1039/d6ra04037c

**Published:** 2026-08-11

**Authors:** Abdul Ghaffar, Safdar Ali Amur, Quratulain Khuhro, Najaf Ali Soomro, Mujahid Mehdi, Jun Liu, Khalid Hussain Thebo, Muhammad Atiq Ur Rehman, Akbar Ali, Ahmed Nadeem

**Affiliations:** a Key Laboratory of Air-Driven Equipment Technology of Zhejiang Province, College of Mechanical Engineering, Quzhou University Quzhou 324000 Zhejiang China 36119@qzc.edu.cn; b Department of Chemistry, Faculty of Natural Science, Government College University Hyderabad 71000 Sindh Pakistan; c Institute of Biochemistry, University of Sindh Jamshoro 76080 Pakistan; d Hangzhou International Innovation Institute, Beihang University China; e Department of Chemistry, Mirpur University of Science & Technology (MUST) A&J Kashmir Pakistan thebokhalid@gmail.com khalidthebo@yahoo.com; f Department of Materials Science and Engineering, Institute of Space Technology Islamabad 44000 Pakistan; g MIIT Key Laboratory of Critical Materials Technology for New Energy Conversion and Storage, State Key Laboratory of Urban Water Resource and Environment, School of Chemistry and Chemical Engineering, Harbin Institute of Technology Harbin 150001 PR China; h Department of Pharmacology and Toxicology, College of Pharmacy, King Saud University Riyadh 11451 Saudi Arabia

## Abstract

Zeolitic imidazolate framework-8 (ZIF-8) is a promising nanocarrier for drug delivery due to its tunable porosity, biocompatibility, and pH sensitivity. In this study, a one-pot room-temperature synthesis strategy was developed to encapsulate polydatin (PD) within ZIF-8 (PD@ZIF-8), followed by chitosan (CS) coating (PD@ZIF-8/CS) for improved stability and sustained release of this drug delivery system. FTIR, UV-vis, XRD, DLS and TGA characterization confirmed the successful synthesis of PD@ZIF-8/CS. SEM micrographs revealed the rhombic dodecahedral particles (85.2 nm) of ZIF-8, slightly roughened polyhedral particles (115.4 nm) of PD@ZIF-8 and aggregated particles (294.8 nm) of PD@ZIF-8/CS, confirming CS shell formation. DLS analysis showed hydrodynamic diameters of 99.3 ± 2.1 nm, 112.8 ± 3.4 nm, and 251.7 ± 4.6 nm for ZIF-8, PD@ZIF-8 and PD@ZIF-8/CS, respectively, with the zeta potential shifting from +12.6 to −3.9 mV after CS coating. The PD@ZIF-8 system achieved an outstanding drug-loading efficiency (91.34%) and exhibited pH-dependent release, reaching 92.5% release at pH 5.0 and 37 °C within 72 h, while the CS coating reduced the burst release by ∼35% and prolonged *t*_0_ from 18.4 h to 38.6 h, following the Korsmeyer–Peppas model (*R*^2^ = 0.991). The PD@ZIF-8/CS composite displayed potent antibacterial activity (inhibition zones: 24.6 ± 0.4 mm for *S. aureus* and 21.2 ± 0.3 mm for *E. coli*; MIC: 6.25 µg mL^−1^), demonstrating a synergistic effect of PD, ZIF-8 and CS. These findings indicate that PD@ZIF-8/CS is a strong and pH-sensitive nanoplatform that can selectively release the drug in the acidic microenvironment of infection sites, suggesting its potential utility as an antibacterial nanoplatform, pending further biological validation.

## Abstract 2

In this study, polydatin-loaded ZIF-8 (PD@ZIF-8) and a chitosan-coated PD@ZIF-8/CS drug delivery system were developed with a high drug loading efficiency (91.34%) and enhanced antibacterial activity.

## Graphical abstract

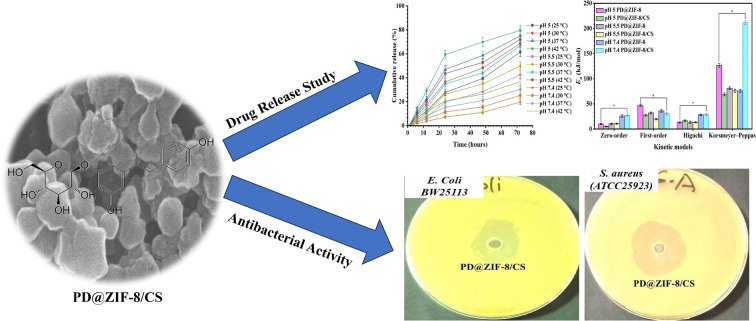

## 1. Introduction

The pursuit of advanced drug delivery systems (DDSs) has revolutionized therapeutics by enhancing drug targeting, improving bioavailability, and reducing adverse effects. Metal–organic frameworks (MOFs), particularly zeolitic imidazolate framework-8 (ZIF-8), are among the most promising nanocarriers due to their high porosity, tunable structure, and chemical stability.^1^ ZIF-8 has a strong sodalite-like structure composed of zinc ions (Zn^2+^) and 2-methylimidazole (2-MIM) that remains stable under physiological conditions; however, it disintegrates in acidic microenvironments such as tumors and lysosomes, enabling pH-responsive release.^2^ However, achieving a high drug loading efficiency without losing the integrity of the ZIF-8 structure is still challenging because too high loading amounts will lead to the partial collapse of the porous architecture and decrease crystallinity, thereby affecting the drug release performance significantly.^3^ Traditional methods, such as solvothermal and microwave-assisted synthesis, produce well-defined crystals but rely on hazardous solvents, high energy inputs, and complex equipment, limiting large-scale production.^4,5^ One-pot room-temperature synthesis offers a greener, more scalable alternative by enabling ZIF-8 formation under mild ambient conditions. This ecofriendly method minimizes waste from solvent, reduces energy demand, and provides precise control over framework properties, making it especially suitable for biomedical applications.^5^

Polydatin (PD), a natural stilbenoid glycoside derived mainly from *Polygonum cuspidatum*, has long been used for treating inflammation, infections, burns, hyperlipemia, and jaundice.^6^ As a stable and bioavailable prodrug of resveratrol, PD offers improved pharmacokinetics compared to its aglycone.^7^ Different studies have shown the therapeutic potential of PD against cancer, cardiovascular and metabolic disorders, neurodegeneration, liver and respiratory diseases, gastrointestinal and rheumatoid conditions, women's health issues, oxidative stress-associated diseases, and antimicrobial resistance.^6,8–10^ However, its clinical application is hindered by poor water solubility, low permeability, and rapid enzymatic or photodegradation, resulting in low systemic bioavailability.^8,11,12^ These limitations highlight the need for advanced delivery systems to improve its stability, solubility, and controlled release at the target site. To address these limitations, we employed ZIF-8 due to its pH-sensitive degradation behavior, which allows it to remain intact at the physiological pH (7.4) but disassemble in acidic environments (pH 5.5–6.5) such as infection sites and intracellular compartments.^5^ This allows site-specific drug release, reduces premature drug leakage and enhances its therapeutic efficiency while protecting the drug from enzymatic degradation and improving its aqueous dispersibility.^13,14^ ZIF-8 has a higher encapsulation efficiency, stability, and pH-triggered release ability than conventional carriers like polymeric nanoparticles, which often leak out the drugs in the carrier and can only carry a limited amount of drugs.^15–17^ Traditional drug-loading strategies for MOFs, such as postsynthetic impregnation, often yield heterogeneous distributions and premature release.^18^ However, the one-pot synthesis approach integrates the drug during framework formation, ensuring strong host–guest interactions, homogeneous loading, and better scalability.^5,13–15^ However, limited studies have demonstrated the one-pot encapsulation of polyphenolic compounds like PD. *In vitro* studies have indicated that ZIF-8 is highly biocompatible, safe, and has low cytotoxicity.^19^ Imidazolate and Zn^2+^ are its degradation products, which have been reported to be metabolized/excreted without significant adverse effects under *in vitro* conditions. However, there is still a need for *in vivo* validation of these studies^5,13,20^ in future works. Further, the biocompatibility of ZIF-8, CS, and PD has been documented in the literature.^5,6,19,21^ To further enhance performance, we introduced a chitosan (CS) coating on PD@ZIF-8. CS is a biocompatible, mucoadhesive polysaccharide with inherent antibacterial activity, acting by disrupting bacterial membranes.^14,22,23^ Previous reports have shown that CS-functionalized MOFs improve colloidal stability, prolong circulation, and facilitate pH-responsive release.^14^ Moreover, CS serves as a semipermeable barrier, enabling sustained or sequential release. Studies have confirmed that such coatings not only enhance bioavailability but also provide synergistic antibacterial effects.^14,21,24^

In this work, we report, for the first time, the one-pot room-temperature synthesis of a PD-loaded ZIF-8 (PD@ZIF-8) system with a CS coating (PD@ZIF-8/CS). The composite harnesses the therapeutic properties of PD, the protective and pH-responsive release characteristics of ZIF-8, and the bio-adhesive and antimicrobial functions of CS. Compared to previously reported ZIF-8-based DDS,^13–15,18^ PD@ZIF-8/CS demonstrates superior MIC values, ampicillin-surpassing antibacterial zones, and a distinctively characterized dual pH- and temperature-responsive release profile, founding it as a scientifically and therapeutically distinct nanoplatform.

## 2. Materials and methods

### 2.1. Chemicals

Polydatin (C_20_H_22_O_8_, 95% pure) and CS with a ≥95% deacetylation degree were purchased from Shanghai Macklin Biochemical Co., Ltd, China. 2-Methylimidazole (99%) and zinc nitrate hexahydrate (Zn(NO_3_)_2_·6H_2_O, 98% pure) were purchased from Aladdin Biochemical Technology Co., Ltd (Shanghai, China). Other chemicals such as ethanol (C_2_H_6_O), phosphate-buffered saline (PBS), acetic acid (CH_3_COOH) and hydrochloric acid (HCl) were of analytical grade.

### 2.2. Synthesis of PD@ZIF-8

PD@ZIF-8 was synthesized through a one-pot room-temperature synthesis method.^5,13,14^ The separate solutions of Zn(NO_3_)_2_·6H_2_O, 2-MIM and PD were prepared by dissolving 0.5 g, 1.5 g, and 10 mg, respectively, in 10 mL of ethanol. In a 250 mL beaker, the Zn(NO_3_)_2_·6H_2_O solution was stirred at 800 revolutions per min (rpm) at 27 °C using a hot-plate magnetic stirrer; thereafter, 2-MIM and PD solutions were added gently. The mixed solution turned milky turbid within a few seconds, indicating the formation of PD@ZIF-8. The reaction mixture was stirred for 1 hour to ensure complete maturation. Similarly, ZIF-8 was prepared without adding PD. The PD@ZIF-8 and ZIF-8 blend solutions were centrifuged at 8000 rpm for 10 min, followed by three washes with absolute ethanol (99.9%, v/v) to remove unreacted precursors, free PD, and any synthesis byproducts. Finally, powdered materials were made using a 12 h freeze-drying process at −55 °C in a vacuum using an Alpha 1-4 LSCbasic lyophilization system. Drug loading encapsulation (DLE) was determined through UV-vis spectroscopy, as reported in earlier works.^13,18^ 1 mg of PD@ZIF-8 was placed in a 2 mL tube. Then, 200 µL of HCl (0.5 M) was added, and the total volume was adjusted to 2 mL with ethanol. The mixture was stirred at 200 rpm for a few minutes at room temperature. The absorbance of the supernatant solution was measured at a wavelength of 313 nm (ref. 25–27) using a UV-vis spectrophotometer (Shimadzu UV-1900i Plus), and the concentration of PD was estimated using the regression equation (*y* = 0.4147*x* + 0.193, *R*^2^ = 0.9971) of the standard calibration curve of PD (Fig. S1) generated with different concentrations ranging 10 µg to 200 µg. DLE was calculated using eqn (1).1
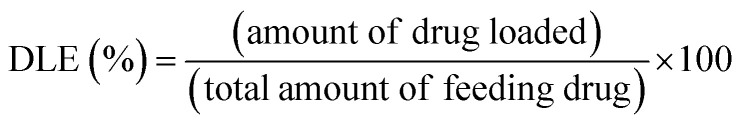


### 2.3. Chitosan coating of PD@ZIF-8

For the CS coating of PD@ZIF-8 to obtain PD@ZIF-8/CS, a 1% CS solution was made in 1% acetic acid by stirring at 1000 rpm overnight at 37 °C. The pH was set to 5.5. Then, a 1 mg mL^−1^ aqueous suspension of PD@ZIF-8 was added to the CS solution in a 1 : 3 ratio, and the blend was stirred at 800 rpm and 27 °C for 24 h.^14^ The PD@ZIF-8/CS was collected using the same procedure as PD@ZIF-8.

### 2.4. Physicochemical characterizations

UV-vis spectroscopic analysis of the aqueous solutions of PD@ZIF-8, PD@ZIF-8/CS, pure ZIF-8, and ethanolic PD and a 1% CS–acetic acid solution was performed using a UV-vis spectrophotometer. The KBr tablet press method was used to record the Fourier transform infrared (FTIR) spectra of all materials in the range of 4000–500 cm^−1^ using a Nicolet 6700 FTIR spectrometer (Thermo Fisher Scientific, Waltham, MA, USA), attached to a DLaTGS detector. Thermogravimetric analysis (TGA) of the materials was performed by placing the samples in platinum pans in a thermal analysis system (Hitachi STA7300); then, they were heated between 34 °C and 800 °C at a rate of 10 °C min^−1^ with a continuous flow of nitrogen gas at a rate of 50 mL min^−1^. To conduct morphological studies, finely ground powder samples were spread evenly across carbon-coated, double-sided, self-adhesive tape, which was attached to brass sample holders used in a scanning electron microscope (SEM). The samples were then coated with a thin layer (∼10 nm) of gold using a gold sputter coater to provide good electrical conductivity and reduce charging artifacts. The HITACHI S-4700 SEM (Japan) instrument was operated at 10 kV under high-vacuum conditions with an accelerating voltage of 10 kV and a working distance of 8 mm. Images were taken at a magnification of 100K. Then, the particle size distribution was evaluated by analyzing the SEM micrographs using the ImageJ 1.54d software (NIH, USA), and results were expressed as mean ± standard deviation. To confirm the elemental composition (C, N, O, and Zn) of ZIF-8, PD@ZIF-8, and PD@ZIF-8/CS and to confirm the successful incorporation of PD and the CS coating, energy-dispersive X-ray analysis (EDAX) elemental mapping was carried out simultaneously with the SEM system using the EDAX software analyzer. For TEM, the samples were dispersed in deionized water at a concentration of 0.5 mg mL^−1^ using moderate sonication. A single drop was applied onto a carbon-covered copper-mesh TEM grid and left to dry overnight at room temperature. The TEM images were acquired at 120 kV on a HITACHI HT7700 (Japan).

The dynamic light scattering (DLS) analysis parameters, such as zeta potential, polydispersity index (PDI), and hydrodynamic diameter (*Z*-average), of the as-prepared materials were studied. 500 µg mL^−1^ powdered samples were thoroughly dispersed in Milli-Q distilled water, followed by filtration (0.22 µm syringe filter) and then subjected to the Malvern Zetasizer (Nano ZSP) system. The zeta potential was recorded in the electrophoretic mode (at 13 °C and 25 °C), while the PDI and *Z*-average were calculated based on the intensity-weighted size dispersion of materials at 173 °C. Moreover, the physicochemical stability of ZIF-8, PD@ZIF-8, and PD@ZIF-8/CS was investigated at different pH, time, and temperature intervals through DLS analysis.

The X-ray diffraction (XRD) patterns of all the samples were analyzed to determine their crystallinity and crystallite size. The diffractograms were recorded using a Bruker D8 Advance powder diffractometer (Germany) equipped with a Cu Kα radiation source (*λ* = 1.5406 Å) operating at 40 kV and 40 mA. Scanning was carried out in the 2*θ* range of 5°–40° with a step size of 0.02° and a scan rate of 10° min^−1^. The degree of crystallinity was calculated from the XRD patterns by comparing the integrated areas of the crystalline peaks to the total area under the curve using eqn (2):2
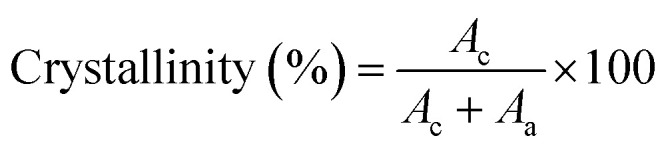
where *A*_c_ is the area of crystalline peaks and *A*_a_ is the amorphous background area.

The crystallite size (*D*) of the dominant diffraction peaks was estimated using the Scherrer equation (eqn (3)):3
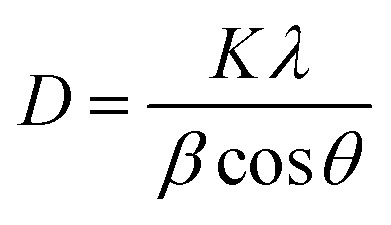
where *K* is the shape factor (0.9), *λ* is the X-ray wavelength (1.5406 Å), *β* is the full width at half-maximum (FWHM, in radians), and *θ* is the Bragg angle of the selected peak.

The background subtraction was done by linear interpolation, and the peak fitting was done by a Gaussian function in OriginPro 2025b to obtain the FWHM value. The 2*θ* = 7.3° (011 plane) peak was chosen for the Scherrer analysis of ZIF-8 and PD@ZIF-8 because the peak at 7.3° had a higher degree of peak overlap than the peak at 10.4° (002 plane) due to the amorphous hump of CS, while the peak overlap was the most significant for PD@ZIF-8/CS at 10.4°.

The specific surface area, pore volume, and pore size distribution of ZIF-8, PD@ZIF-8 and PD@ZIF-8/CS were determined by nitrogen adsorption–desorption measurements at −195 °C using a Micromeritics ASAP 2460 analyzer. The samples were degassed for 12 h under vacuum at 150 °C before analysis to eliminate the water and impurities on the surface. During the adsorption–desorption cycle, the relative pressure (*P*/*P*_0_) was gradually raised from 0 to 1. The Brunauer–Emmett–Teller (BET) method was used to calculate the specific surface area from the adsorption isotherm data. The amount of N_2_ adsorbed at *P*/*P*_0_ = 0.99 was used to calculate the total pore volume. The Barrett–Joyner–Halenda (BJH) method was used for pore size distribution analysis while considering the desorption branch of the isotherm. The average pore diameter was estimated by the following formula: pore diameter = 4*V*/*A*, where *V* is the total pore volume and *A* is the BET surface area.

### 2.5. *In vitro* drug release

The drug release from PD@ZIF-8 and PD@ZIF-8/CS was studied at different pH levels, *i.e.*, 5, 5.5, and 7.4, at 25 °C, 30 °C, 35 °C, 37 °C, and 42 °C for 1–72 h. The drug release medium consisted of 20 mM PBS with 40% ethanol (v/v) and the drug stabilizer Tween-80 (0.5%, v/v). To maintain the sink conditions for the release study, 40% ethanol was added to ensure adequate solubility of PD, which has limited water solubility (∼0.1 mg mL^−1^), during the 72 h release study. The method was quite similar to that used in the release studies of previously reported poorly water-soluble polyphenolic drugs that were encapsulated in MOF-based carriers.^13,15^ An accurately weighted 10 mg of PD@ZIF-8 and PD@ZIF-8/CS was dispersed in the drug release medium, which was then packed into dialysis bags with a molecular weight of 12–14 kDa. These were shifted to beakers containing 50 mL of the medium system of each pH. Finally, these assemblies were magnetically stirred at 200 rpm. Thus, at each defined time break, 1 mL of the media was pipetted and replaced with the same volume, and the absorbance of free PD was recorded spectrophotometrically at a wavelength of 313 nm.^25–27^ The concentration of released PD was determined using the standard calibration curve of PD (Fig. S1). The cumulative drug release^13^ was calculated using eqn (4):4
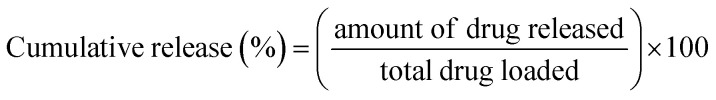


The stability of PD was also evaluated under different pH conditions (pH 7.4, pH 6.5, pH 5.5, and pH 5.0) to simulate physiological and pathological environments, such as normal bloodstream conditions (pH 7.4), tumor microenvironments (pH 6.5 and pH 5.5), and lysosomal/endosomal compartments (pH 5.0). The study was conducted by incubating PD in PBS at the respective pH values, followed by spectrophotometric analysis using the characteristic absorption peak of PD (313 nm). The percentage of remaining PD at each time point at each pH was calculated using the standard calibration curve.

*In vitro* drug release profiles were analyzed using four established kinetic models: zero-order, first-order, Higuchi, and Korsmeyer–Peppas models^13,14^ (SI). The zero-order model assumes a constant release rate independent of the drug concentration (*Q*_*t*_ = *Q*_0_ + *k*_0_*t*), while the first-order model describes concentration-dependent release (log *Q*_*t*_ = log *Q*_0_ − *k*_1_*t*/2.303). The Higuchi model is based on Fickian diffusion in a homogeneous matrix (*Q*_*t*_ = *k*_H_√*t*), and the Korsmeyer–Peppas model (*M*_*t*_/*M*_∞_ = *k*_KP_*t*^*n*^) accounts for both Fickian and non-Fickian release, where the release exponent, *n*, indicates the underlying mechanism. Model fitting was performed at different pH values (5.0, 5.5, and 7.4) and temperatures (25 °C, 30 °C, 37 °C, and 42 °C), and regression coefficients (*R*^2^) were used to evaluate the goodness of fit (Table S1). To assess the temperature dependence of release kinetics, Arrhenius analysis was applied to the rate constants (*k*) obtained from each model, and Arrhenius plots (Fig. S2 and S3) were generated for each kinetic model and pH condition. Finally, the activation energy (*E*_a_) values were calculated and compared to depict the energy barrier and release mechanism under varying physiological environments (SI).

### 2.6. Antibacterial evaluation of materials against *E. coli* and *S. aureus*

The antibacterial efficacy of the synthesized materials was systematically assessed against *Escherichia coli* (BW25113) and *Staphylococcus aureus* (ATCC25923) using the agar well diffusion assay on Luria–Bertani (LB) agar plates.^5,13,14^ Sterile 60 mm Petri dishes were prepared by pouring molten LB agar, followed by surface inoculation with standardized bacterial suspensions. Wells of 6 mm diameters were aseptically punched into the solidified agar using a sterile metal cork borer. Each well was loaded with 50 µL of test solutions from stock solutions of PD@ZIF-8, PD@ZIF-8/CS, ZIF-8, PD and ampicillin (1 mg mL^−1^ in dimethyl sulfoxide, 5% v/v). The plates were incubated at 37 °C for 24 h, and antibacterial zones of inhibition were measured.

Minimum inhibitory concentrations (MICs) were determined using a standard broth microdilution method in a 96-well plate format. Bacterial suspensions (OD_600_ = 0.5) were added to wells containing serial dilutions of the test materials (15, 25, 35, and 50 µg mL^−1^) and incubated at 37 °C for 24 h, and OD_600_ was recorded. The MIC was defined as the lowest concentration at which no observable bacterial growth occurred. Furthermore, a time-kill assay was performed to investigate the bactericidal kinetics and concentration-dependent effects of PD@ZIF-8 and PD@ZIF-8/CS against the tested bacteria. For this, bacterial cultures were exposed to varying concentrations of the test materials in LB broth and incubated at 37 °C with continuous agitation. The optical density at 600 nm (OD_600_) was recorded at hourly intervals over a 24 h period to monitor bacterial growth dynamics.

To determine the disruption of the bacterial membrane, the release of intracellular nucleic acid from cell suspensions of *S. aureus* and *E. coli* was determined spectrophotometrically.^5^ In brief, recently cultured bacterial cells in the logarithmic phase were centrifuged, washed with PBS, and once again resuspended in PBS. The appropriate bacterial suspension was then cocultured with PD@ZIF-8 and PD@ZIF-8/CS at 1× and 2× MICs. Only PBS was present in the control group. All assays were incubated at 37 °C with shaking, and sample aliquots were taken at 1, 3, 6, and 12 h. The absorbance of the supernatant of each aliquot at 260 nm was determined using a UV-vis spectrophotometer. The absorbance was measured and recorded against the corresponding blank for each assay (PBS and a normal bacterial cell suspension).

### 2.7. Data analysis

Each experiment was carried out three times (*n* = 3), and data are presented as mean ± standard deviation (SD). Microsoft Excel 2016 and OriginPro 2025b were used to process the data, such as UV-vis spectroscopy, FTIR spectroscopy, TGA, SEM, XRD, DLS, drug release assays, and antibacterial assays. For statistical analysis, one-way ANOVA was applied, followed by Tukey's honest significant difference (HSD) *post hoc* test with a significance level set at *p* < 0.05.

## 3. Results and discussion

### 3.1. Physicochemical characterizations

**Fig. 1:**
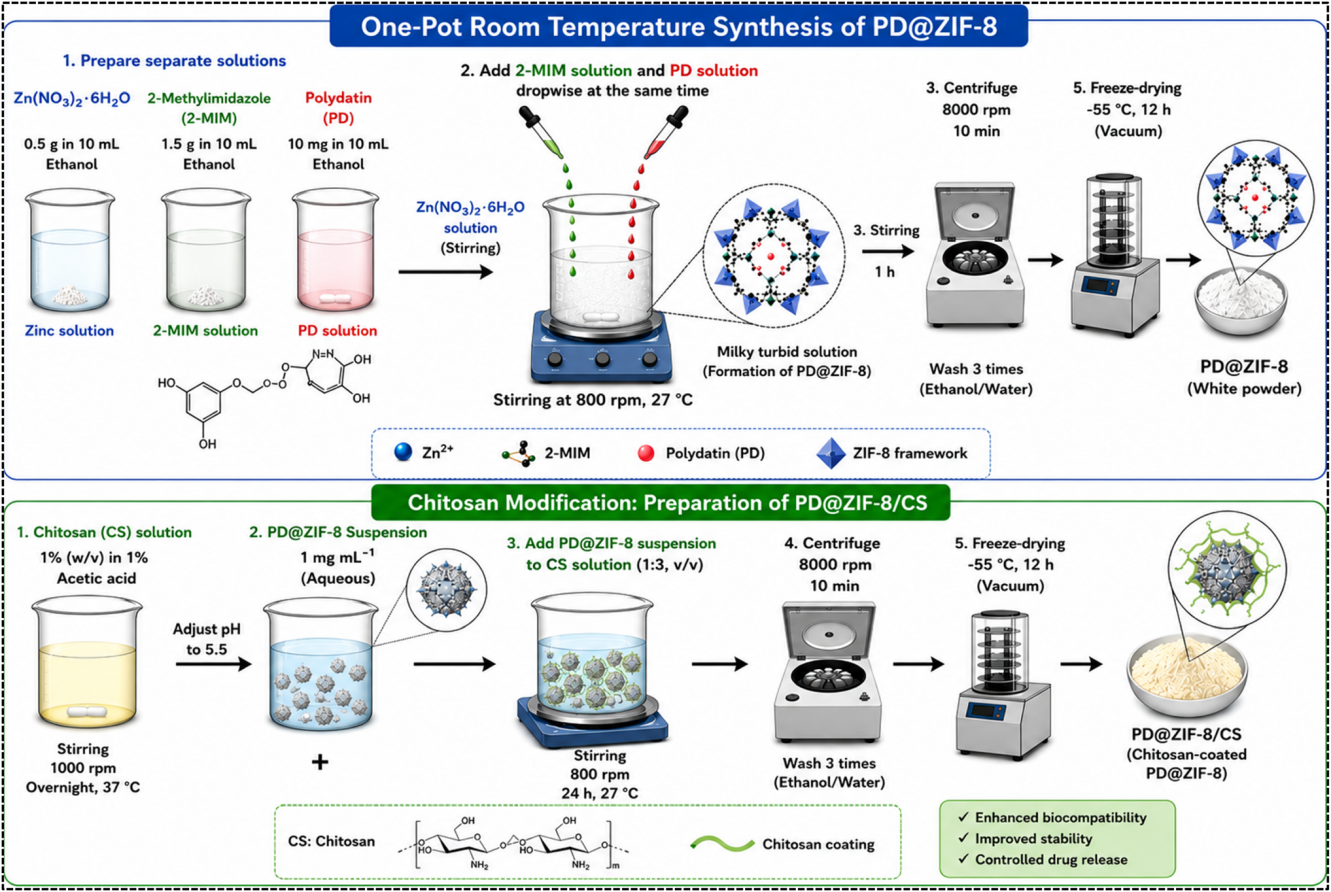
One-pot room-temperature synthesis of PD@ZIF-8, followed by CS coating to obtain PD@ZIF-8/CS with enhanced stability and controlled release.

In this study, a one-pot room-temperature synthesis approach^13–15,18^ was employed to encapsulate PD into the ZIF-8 framework, yielding PD@ZIF-8 (Fig. 1). The PD@ZIF-8 formulation achieved a DLE of 91.34%, confirming the effective encapsulation of PD within the porous ZIF-8 matrix.^5^ Furthermore, to enhance the stability and therapeutic performance of PD@ZIF-8, a CS coating was introduced to form PD@ZIF-8/CS (Fig. 1). The DLE of PD@ZIF-8 was slightly lower than that of Glab@ZIF-8 (glabridin, ≈98.7%)^13^ but surpassed that of Mag@ZIF-8 (magnolol, 89.67%), PHY@ZIF-8 (physcion, 88%), GPS@ZIF-8 (gentiopicroside, 84%), and ZIF-8@CCM (curcumin, 82.76%).^14,15,18,28^ The high DLE of PD@ZIF-8 (91.34%) can be explained by the multiple hydroxyl groups in the PD molecule, which can form strong hydrogen bonding interactions with the Zn–N coordination sites and imidazolate linkers of the ZIF-8 framework during the one-pot synthesis. These hydrogen bonding interactions result in favorable host–guest encapsulation, as seen for Glab, which has several hydroxyl functionalities and therefore a high DLE.^13^

The CS biopolymer layer, which is rich in amino and hydroxyl groups,^14^ is responsible for reinforcing the structural integrity and resistance to early degradation, as indicated by the DLS stability data, in which PD@ZIF-8/CS displayed stable size growth (214–259 nm) with PDI values consistently below 0.5 for a period of 2 weeks, whereas PD@ZIF-8 without the CS biopolymer coating showed greater dimensional growth instability (Fig. 6a). The pH-responsive charge reversal of PD@ZIF-8/CS from −3.88 mV at pH 7.4 to +5.9 mV at pH 4 (Fig. 6f) further indicates its stimulus-responsive behavior.^14^ Furthermore, the CS coating significantly reduced the burst release by ∼35% and increased the *t*_50_ from 18 h to 38 h (Fig. 10b), which shows pH-responsive controlled release. These results are similar to those reported for other systems that were made with CS coating, which resulted in enhanced colloidal stability, minimized burst release, and allowed for pH-dependent drug release.^14,21,22^

**Fig. 2:**
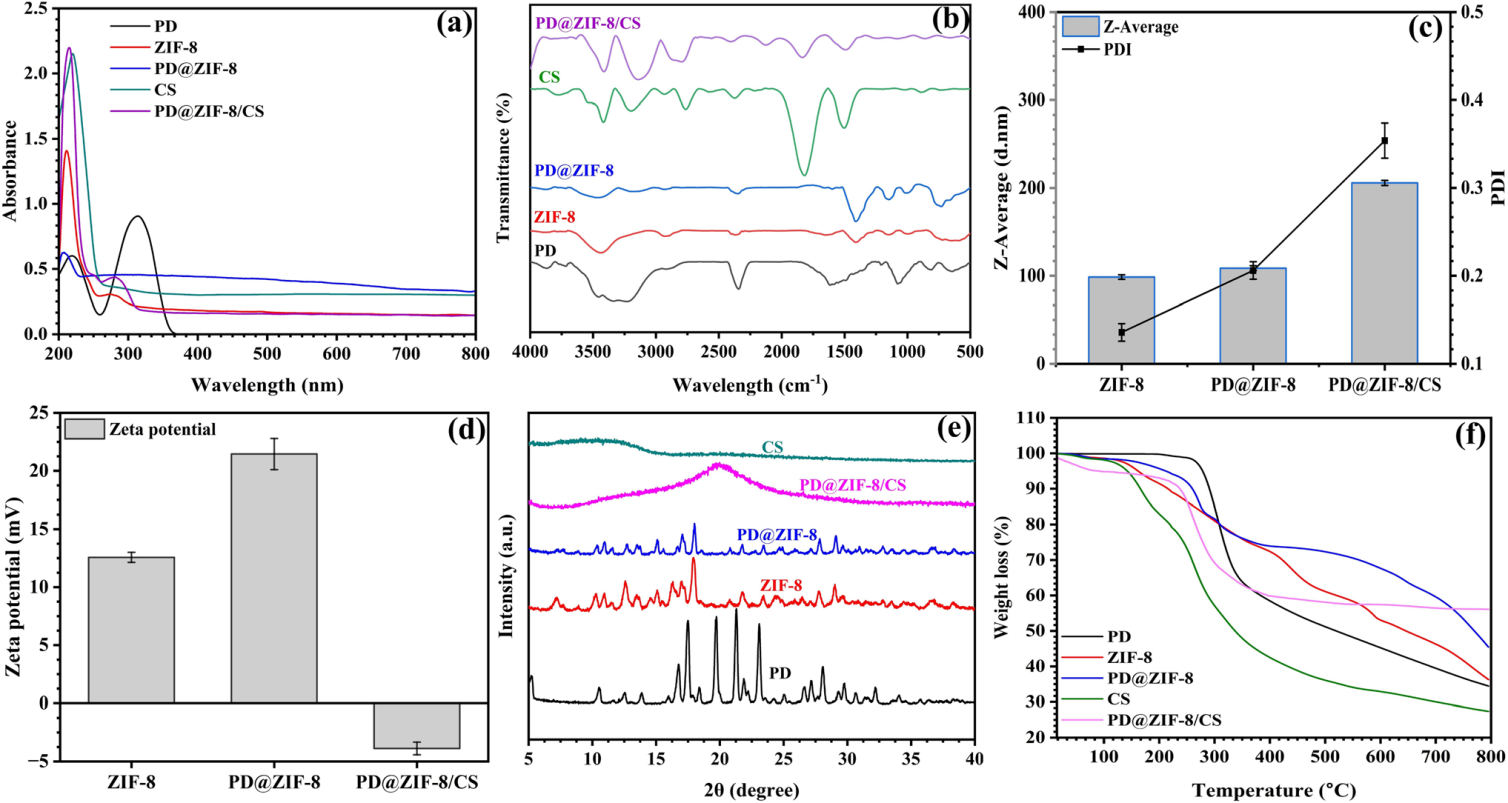
Physicochemical characterization of the materials: (a) UV-vis spectra, (b) FTIR spectra, (c) DLS particle size distributions and PDIs, (d) zeta potentials, (e) XRD patterns, and (f) TGA thermograms of PD, ZIF-8, PD@ZIF-8, and PD@ZIF-8/CS.

The UV-vis spectra of the pure PD, CS, ZIF-8 and PD@ZIF-8 and PD@ZIF-8/CS composites revealed distinct electronic transitions, confirming successful encapsulation and surface modification (Fig. 2a). Pure PD exhibited two characteristic absorption peaks at 220 nm and 313 nm,^25,29^ corresponding, respectively, to the π → π* transition of the aromatic rings and the extended conjugated *trans*-stilbene system of the aglycone moiety, which are typical features of PD and related stilbenoids.^25–27^ CS displayed a single absorption peak at 221 nm, attributed to the n → π* transitions of carbonyl and amine groups associated with residual *N*-acetylated units.^30^ The ZIF-8 spectrum showed a band centered at 212 nm, which can be ascribed to the π → π* transitions of the imidazolate ligands and the ligand-to-metal charge transfer between the Zn^2+^ centers and imidazole rings, consistent with previously reported ZIF-8 spectra.^5,13,14^ After the encapsulation of PD into ZIF-8, a hypsochromic shift from 220 nm to 207 nm was found, along with the disappearance of the 313 nm band. Such a spectral change does not mean the absence of PD, but rather its successful encapsulation within the ZIF-8 pores.^15^ The presence of PD is indicated by the 313 nm band, which is completely suppressed due to the confinement of PD within the nanoporous framework, reducing its π-conjugation and changing its electronic environment *via* coordination to Zn^2+^ sites and π–π stacking with imidazolate linkers. The disappearance of the drug-specific UV peak is consistent with the results of previously published ZIF-8-based DDS, where a similar disappearance of drug-specific UV peaks was used as evidence of successful encapsulation.^13–15,18^ The blue shift and band quenching are attributed to strong host–guest interactions and restricted π-conjugation upon encapsulation within MOFs.^13,14^ When the composite was coated with CS, the absorption band slightly red-shifted to 214 nm, implying hydrogen bonding and electrostatic interactions between the polymer matrix and the ZIF-8 surface, which partially altered the local polarity around PD.^14^ These spectral modifications collectively confirm the successful loading of PD into the ZIF-8 framework and the subsequent formation of the PD@ZIF-8/CS hybrid with a stable polymeric coating.

The FTIR spectra of PD, CS, ZIF-8, PD@ZIF-8, and PD@ZIF-8/CS were analyzed to confirm the stepwise assembly of the synthesized drug delivery system (Fig. 2b). PD exhibited a broad O–H band at ∼3365 cm^−1^, aliphatic C–H stretches at 2931 and 2844 cm^−1^, and a strong carbonyl/aromatic absorption at 1644 cm^−1^. Aromatic skeletal bands were visible at 1604, 1510, and 1450 cm^−1^; glycosidic C–O–C vibrations appeared at 1274, 1147, 1084, and 1028 cm^−1^; the sharp peak at 996 cm^−1^ corresponded to glycosidic bonds; and the bands at 850–650 cm^−1^ indicated aromatic C–H bending (Fig. S4a and Table S3). These assignments confirm the polyphenolic glycoside structure of PD.^11,31,32^ CS showed a broad O–H/N–H stretch at ∼3435 cm^−1^, a C–H stretch at 2931 cm^−1^, and amide I (∼1656 cm^−1^) and amide II (∼1590 cm^−1^) bands. Partially deacetylated CS^33^ exhibited characteristic peaks at ∼1820 cm^−1^ (C=O stretching) and ∼1504 cm^−1^ (N–H bending) (Fig. S4b and Table S3). The absorption at 883 cm^−1^ was associated with saccharide ring deformation, while the one at 724 cm^−1^ was assigned to C–H out-of-plane bending. The FTIR spectrum of ZIF-8 (Fig. S4c and Table S3) revealed the expected vibrational features of the 2-methylimidazolate ligand coordinated to Zn^2+^. The distinct absorption at ∼420 cm^−1^ was attributed to Zn–N stretching, the fingerprint vibration of ZIF-8.^5^ Aromatic and aliphatic C–H stretching bands were observed at ∼3134, 2929, and 2851 cm^−1^. The strong absorption at 1584 cm^−1^ originated from the C=N stretching of the imidazole ring, while additional imidazole skeletal vibrations appeared at 1450–1380 cm^−1^.^5,13,15,18^ In-plane bending and C–N vibrations were detected at 1274, 1147, 1071, and 1042 cm^−1^, while the peaks near 995–920 cm^−1^ were assigned to imidazole ring deformation.^5^ Aromatic C–H out-of-plane bending was also observed in the 800–650 cm^−1^ region. The absence of broad N–H stretches (3300–3500 cm^−1^) confirmed the deprotonation of the imidazole ligand upon coordination with Zn^2+^.^5,34,35^ In PD@ZIF-8, the characteristic ZIF-8 bands (Zn–N at 420 cm^−1^ and C=N at 1584 cm^−1^) were retained, while PD-related peaks (broad O–H at 3370–3320 cm^−1^, C–H at 2931/2844 cm^−1^, C=O at 1644 cm^−1^, and glycosidic C–O at 1147–1028 cm^−1^ (Fig. S4d and Table S3)) were also present but with reduced intensity and slight shifts. These spectral changes indicate hydrogen bonding between PD hydroxyl groups and the imidazolate/Zn^2+^ sites, confirming successful encapsulation.^5,13,15,18^ PD@ZIF-8/CS (Fig. S4e and Table S3) showed the combined features of all three components: a broadened O–H/N–H stretch at 3435–3300 cm^−1^, amide I (∼1650 cm^−1^) and amide II (∼1590 cm^−1^) bands overlapping with imidazole C=N, and strong glycosidic C–O–C absorptions at 1084 and 1028 cm^−1^.^14,36^ The ZIF-8 Zn–N band (∼420 cm^−1^) remained, confirming framework stability. The PD peaks at 1644 and 996 cm^−1^ were retained but attenuated, reflecting drug confinement within the pores and shielding by the CS layer. Overall, FTIR analysis verifies the stepwise formation of PD@ZIF-8 and PD@ZIF-8/CS, with PD successfully encapsulated in the ZIF-8 matrix and the CS coating providing biocompatibility, stability, and potential for sustained release.^14,36^

The ZIF-8 solubilized in water exhibited an average hydrodynamic diameter of 98.5 ± 2.5 nm with a low PDI (0.136 ± 0.01), as shown in Fig. 2c, indicating a uniform and monodisperse particle population.^5,13,14^ Upon PD encapsulation, the particle size slightly increased to 108.6 ± 2.8 nm, with a corresponding rise in the PDI (0.21 ± 0.01), confirming successful PD loading within the ZIF-8 framework.^13,15,18^ Notably, the CS coating further enlarged the hydrodynamic diameter to 205.8 ± 2.9 nm and increased the PDI (0.35 ± 0.02), which can be attributed to the formation of a polymeric shell around the drug-loaded MOFs.^14^ ZIF-8 displayed a moderately positive surface charge (+12.57 ± 0.4 mV; Fig. 2d), consistent with previous reports,^5,13^ and it increased after PD loading (PD@ZIF-8, +21.45 ± 1.34 mV; Fig. 2d) due to the contribution of the protonated functional groups of drugs.^13–15^ The enhanced positive surface potential renders these materials highly suitable for antibacterial applications.^5,13,14^ Interestingly, a charge reversal was observed after CS coating, with the surface potential shifting to −3.88 ± 0.54 mV (in water at normal pH; Fig. 2d), indicating the successful deposition of the CS layer.^14^ This inversion of charge not only confirms the presence of CS but also suggests the improved colloidal stability and biocompatibility of the final formulation.^14^

Fig. 2e shows the comparative XRD patterns of the pure PD, CS, ZIF-8, PD@ZIF-8, and PD@ZIF-8/CS nanocomposites. The XRD pattern of pure PD (Fig. S5a) exhibited a series of intense and sharp diffraction peaks at 2*θ* = 10.54°, 11.41°, 13.83°, 14.98°, 15.85°, 17.46°, 18.37°, 19.40°, 20.97°, 22.08°, 23.30°, 24.75°, 26.09°, 27.18°, 28.43°, 29.93°, 31.19°, 32.40°, 33.92°, 35.29°, and 36.98°, confirming the highly crystalline nature of PD. These reflections correspond to the ordered packing of PD molecules stabilized by π–π stacking and intermolecular hydrogen bonding among phenolic –OH and glucosidic –OH groups. The sharpness and high intensity of these reflections are consistent with previously reported crystalline polymorphs of PD obtained from ethanolic and aqueous systems.^11,37,38^ Quantitatively, PD exhibited a crystallinity of 66.98% with an average crystallite size of 37.6 nm (Table S4), confirming its long-range order and high purity. The XRD pattern of CS (Fig. S5b) displayed two broad characteristic reflections centered at approximately 2*θ* = 9.84° and 19.84°, with no other distinct peaks across the 5°–40° range, indicating its semicrystalline or partially amorphous structure.^33,39^ The low-angle reflection (∼9.8°) represents the hydrated crystalline form (CS crystal I), while the broad reflection at ∼20° arises from rearrangements among polymeric chains *via* hydrogen bonding between –NH and –OH groups.^14^ The absence of sharp peaks confirms the limited long-range order typical of native CS. ZIF-8 (Fig. S5c) showed intense and well-defined diffraction peaks at 2*θ* = 7.3°, 10.4°, 12.7°, 14.8°, 16.4°, 18.0°, 24.4°, 26.8°, and 29.6°, indexed to the (011), (002), (112), (022), (013), (222), (233), (134), and (044) planes of the sodalite-type cubic ZIF-8 framework (space group *I*4̄3*m*), respectively.^5,13,14^ These peaks match the simulated XRD pattern from the Cambridge Structural Database (CCDC no. 602542) and JCPDS no. 00-062-1030. The strong reflections confirm the formation of a highly crystalline ZIF-8 phase built from Zn^2+^ ions tetrahedrally coordinated with 2-MIM linkers. The absence of extra peaks indicates phase purity, consistent with previous reports.^34,40,41^ The ZIF-8 sample showed a crystallinity of 68.56% and an average crystallite size of 42.3 nm (Table S4), typical for nano-sized ZIF-8 frameworks.

After PD encapsulation (PD@ZIF-8; Fig. S5d), the system retained the characteristic ZIF-8 peaks at 2*θ* = 7.24°, 10.37°, 12.07°, 16.83°, and 18.04°, but with visibly reduced intensity and peak broadening compared to ZIF-8 (2*θ* = 7.06°, 10.39°, 12.56°, 16.29°, and 17.90°). The broadening was most clearly observed at 2*θ* = 7.3° and 12.1°, a sign of partial loss of the long-range crystalline order in the presence of PD in the ZIF-8 framework. The disappearance of PD-specific crystalline peaks confirms that PD was successfully encapsulated within the nanoporous framework, rather than merely adsorbed on the surface.^13–15,42^ The attenuation in intensity and peak broadening suggest a partial loss of crystallinity, reflecting strong host–guest interactions between PD and the Zn–imidazolate network through hydrogen bonding and π–π stacking. The calculated crystallinity (72.61%) and crystallite size (31.8 nm), as presented in Table S4, indicate the partial stabilization of the ZIF-8 framework upon PD confinement. These results align with previous findings for drug-loaded ZIF-8 systems.^13,15,43,44^ For the PD@ZIF-8/CS composite (Fig. S5e), the XRD pattern retained the major ZIF-8 reflections (2*θ* = 7.3°, 10.4°, and 12.7°) but with markedly reduced intensity, while a broad hump centered at 2*θ* = 19.9° appeared, characteristic of CS.^39^ This pattern confirms the successful coating of crystalline PD@ZIF-8 nanoparticles with a semicrystalline CS matrix, forming a hybrid material with diminished long-range order.^14^ The crystallinity decreased slightly to 75.90%, while the crystallite size decreased to 25.9 nm (Table S4), implying that the CS coating restricts crystal growth and induces surface amorphization.^14,21,24,39^ Such features are consistent with reports on the ZIF-8/CS composites, confirming that polymeric coatings attenuate the diffraction intensity but preserve the fundamental sodalite topology.^14,45^ Overall, the comparative XRD studies validate the successful encapsulation of PD into the ZIF-8 framework and the subsequent CS coating, leading to the formation of a nanocrystalline hybrid (PD@ZIF-8/CS) with preserved framework integrity, a reduced particle size, and a slightly diminished crystallinity, ideal for pH-responsive and sustained drug delivery applications.

The thermal stability and degradation behavior of PD, CS, ZIF-8, PD@ZIF-8, and PD@ZIF-8/CS are shown in Fig. 2f. Pristine PD exhibited a two-step thermal degradation pattern (Fig. S6a). The initial minor weight loss of 0.94–1.49% was noticed below 266 °C. A major weight loss of 30.8% occurred between 266 °C and 336 °C, corresponding to the cleavage of glycosidic bonds and the oxidative degradation of the stilbene moiety.^46^ Beyond 336 °C, PD continued to degrade gradually, reaching a total mass loss of 65.5% with a residue of 34.5% at 793 °C, suggesting the formation of carbonaceous char due to aromatic stabilization.^47^ CS showed a multistep decomposition profile (Fig. S6b). The first event was below 100 °C (≈1.4% loss), followed by a second degradation step between 138 °C and 224 °C (≈10.5% loss) due to the cleavage of glycosidic linkages and the deacetylation of the polymer chain.^14^ The main degradation occurred from 224 °C to 581 °C (≈40.6% loss), associated with polymer backbone depolymerization and the oxidative scission of glucosamine units.^39,48^ The overall weight loss of 72.5% and a 27.5% residue at 789 °C indicate significant carbonization typical of CS-based systems. ZIF-8 demonstrated enhanced thermal resistance (Fig. S6c), undergoing only 1.1% mass loss below 150 °C and a gradual decomposition starting above 300 °C, culminating in major mass losses between 300 °C and 500 °C (≈17.4%), corresponding to the breakdown of the 2-MIM linker and the collapse of the Zn–N coordination framework.^5^ The total weight loss of ≈63.5% and a residue of 36.5% confirm the partial retention of the inorganic ZnO phase after complete combustion.^13–15^

The encapsulation of PD into ZIF-8 (PD@ZIF-8) altered the thermal degradation profile significantly (Fig. S6d). The initial weight loss (∼1.5%) below 100 °C corresponded to surface moisture removal,^15,18^ followed by a major stage at ∼280 °C (≈14.8% loss) and a subsequent moderate degradation between 395 °C and 650 °C (≈17.1% loss). The overall reduction in the total mass loss to ≈54.4%, along with a ≈45.6% residue, suggests that the confinement of PD within the ZIF-8 cavities restricts volatilization and suppresses direct oxidation, indicating enhanced thermal stability, similar to previously reported studies.^5,13,14^ The PD@ZIF-8/CS composite exhibited the highest thermal resistance among all samples (Fig. S6e), showing an overall mass loss of ≈43.8% and a residue of ≈56.2% at 793 °C. The initial stage (<100 °C, ≈4.8%) was due to surface moisture evaporation, followed by successive degradation events at 226–394 °C (≈23.9%) corresponding to the decomposition of CS and partial PD release. The latter stages (394–728 °C, ≈15%) represent the degradation of the ZIF-8 framework and the carbonization of the biopolymer matrix. The significant residue formation reflects the synergistic stabilization between CS and ZIF-8, where the polysaccharide coating acts as a thermal barrier and promotes char formation.^49,50^ The trend in thermal stability follows the order: PD < CS < ZIF-8 < PD@ZIF-8 < PD@ZIF-8/CS, confirming that ZIF-8 encapsulation and CS coating synergistically improve the thermal robustness of the composite, which is crucial for maintaining drug integrity during processing and delivery.^14^

**Fig. 3:**
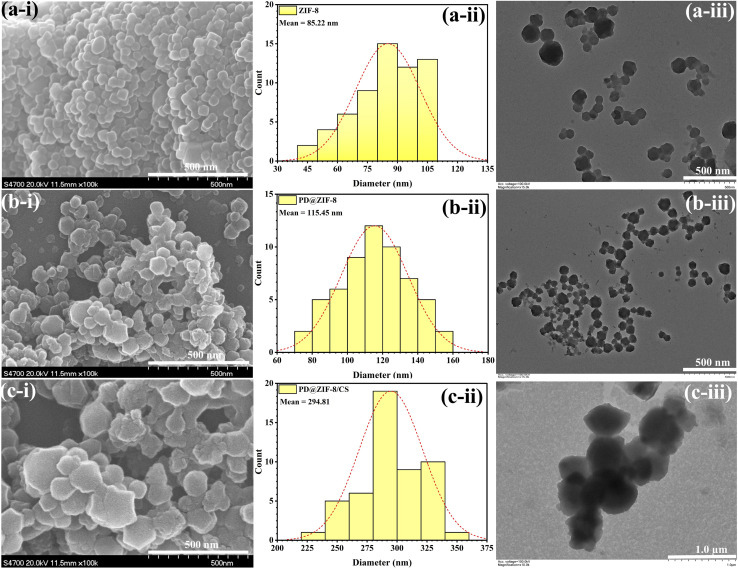
SEM micrographs, particle size distributions, and TEM images of (a-i–a-iii) ZIF-8 and (b-i–b-iii) PD@ZIF-8 and (c-i–c-iii) PD@ZIF-8/CS nanocomposites.

The morphology of ZIF-8, PD@ZIF-8, and PD@ZIF-8/CS was investigated using SEM and TEM. The ZIF-8 showed a typical rhombic dodecahedral morphology (Fig. 3a-i and iii) and exhibited an average dimension of 85.22 nm (Fig. 3a-ii) with a well-monodispersed distribution.^5^ Similar to previous studies,^13–15^ PD@ZIF-8 maintained the polyhedral structure but with rougher surfaces and a slight distortion of the morphology, displaying moderate aggregation after PD loading (Fig. 3b-i and iii). The mean diameter increased to 115.45 nm (Fig. 3b-ii), reflecting a ∼35% size enlargement due to pore occupancy.^13–15,18^ However, the mean size of PD@ZIF-8/CS showed a significant increase to 294.81 nm upon CS coating, indicating the complete coverage of the particles with a uniform polymeric shell and the presence of aggregated and rougher particles with partially obscured polyhedral edges (Fig. 3c-i). In addition, the TEM image of PD@ZIF-8/CS revealed the existence of larger, diffuse, blurry-edged particles, which directly proved that the CS coating was successfully realized in PD@ZIF-8/CS particles (Fig. 3c-iii).^14^

Of particular interest, the absolute size determined from the DLS data and the SEM data was different for identical materials. The DLS measures and reports the hydrodynamic diameter, including the solvation shell in an aqueous suspension, while SEM measures the actual physical diameter of the dried particles.^51^ While SEM gave a larger size difference between the particles (85.2 nm *vs.* 115.4 nm), which more typically reflects the effect of the PD encapsulation on the physical size of the particles, the less pronounced change in the hydrodynamic size by the DLS (98.5 nm *vs.* 108.6 nm) reflects the greater influence of the surface charge and the solvation effect when these reflect the size of the particles in a suspension. This showcased DLS–SEM failure for MOF-based nanoparticles is not an obstacle to the integration of the drugs onto these small structures, as successfully done in numerous studies.^13–15^

**Fig. 4:**
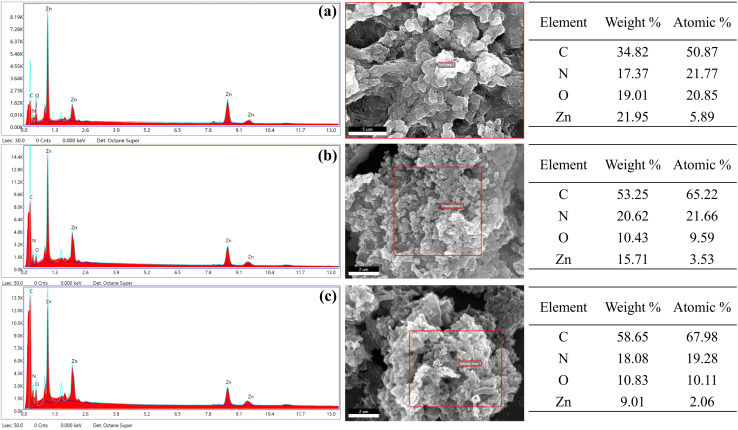
EDX elemental analysis of (a) ZIF-8, (b) PD@ZIF-8, and (c) PD@ZIF-8/CS.

The elemental composition of ZIF-8, PD@ZIF-8, and PD@ZIF-8/CS was further confirmed by elemental analysis (EDX). ZIF-8 (Fig. 4a) showed the characteristic elements C (34.82%), N (17.37%), O (19.01%), and Zn (21.95%), consistent with its Zn^2+^ and 2-MIM framework composition.^13^ After PD encapsulation, the PD@ZIF-8 (Fig. 4b) exhibited a 15.71% weight loss in Zn and a remarkable weight gain in C (53.25%), which is likely due to the encapsulation of PD molecules in the pores of ZIF-8, indicating the successful loading of the PD molecules.^13,14^ The PD@ZIF-8/CS (Fig. 4c) showed a slight increase in the C content (58.65%) and a significant decrease in the Zn weight% (9.01%) due to the contribution of the C-rich CS polysaccharide chains on the surface of the particle.^14,24,39^ The progressive decrease in the weight% of Zn from ZIF-8 (21.95%) to PD@ZIF-8 (15.71%) to PD@ZIF-8/CS (9.01%) clearly shows successful PD encapsulation and CS coating.^14,43^

**Fig. 5:**
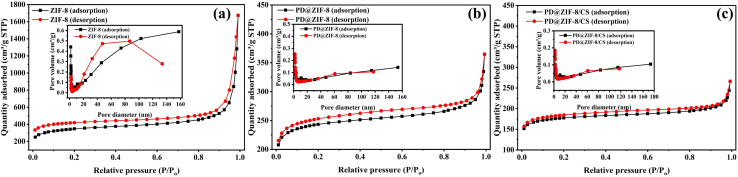
Nitrogen adsorption–desorption isotherms and corresponding pore size distribution curves (insets) of (a) ZIF-8, (b) PD@ZIF-8, and (c) PD@ZIF-8/CS.

In order to further investigate the porous structure and confirm the successful drug encapsulation and coating with CS, N_2_ adsorption/desorption analysis was carried out for ZIF-8, PD@ZIF-8, and PD@ZIF-8/CS. The ZIF-8 showed the typical type I isotherm features of a microporous material, with a BET surface area of 1289.0 m^2^ g^−1^, a micropore area of 1221.0 m^2^ g^−1^, and a micropore volume of 0.468 cm^3^ g^−1^ (Fig. 5a), which are characteristic of the high microporosity of ZIF-8.^5,13,14^ The successful encapsulation of PD molecules inside the pores of ZIF-8 was confirmed by the shift towards a type IV isotherm and the decreased BET surface area of 742.0 m^2^ g^−1^ (Fig. 5b), which led to a significant reduction in the micropore area (678.0 m^2^ g^−1^) and micropore volume (0.261 cm^3^ g^−1^). The high DLE of 91.34% is in excellent agreement with the ∼42% reduction in the surface area.^13^ The BET surface area and micropore volume of PD@ZIF-8/CS (534.0 m^2^ g^−1^ and 0.184 cm^3^ g^−1^, respectively, as shown in Fig. 5c) were further decreased, likely due to the polymeric shell of CS covering the outside surface, precluding some pores from being entered.^14^ The BJH average pore diameter increased gradually with the introduction of the drug in the MOF as well as the modification of the CS, from 2.24 nm (ZIF-8) to 4.62 nm (PD@ZIF-8) and 5.84 nm (PD@ZIF-8/CS), as found in recently reported systems for drug delivery based on MOFs.^52,53^ The gradual reduction in the surface area and micropore volume of ZIF-8, PD@ZIF-8 and PD@ZIF-8/CS, respectively, supports the drug encapsulation and surface coating in a step-by-step manner, which is aligned with the results of FTIR, XRD, DLS, SEM, and TEM characterizations.

### 3.2. DLS stability analysis under different conditions

**Fig. 6:**
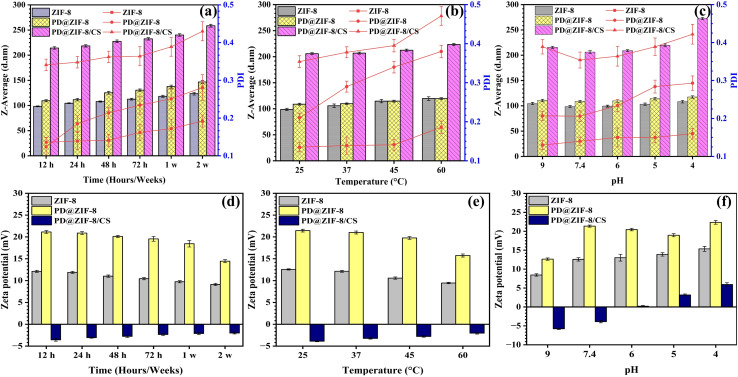
DLS analysis of the ZIF-8, PD@ZIF-8, and PD@ZIF-8/CS nanocomposites showing variations in the hydrodynamic size and distribution under different conditions: (a and d) various time intervals, (b and e) different temperatures, and (c and f) varying pH levels.

The structural stability of ZIF-8, PD@ZIF-8, and PD@ZIF-8/CS was systematically evaluated by DLS under prolonged incubation, varying temperatures, and different pH values. Over the different time intervals (time stability; Fig. 6a and d), ZIF-8 exhibited a gradual increase in the hydrodynamic size from ∼98 nm at 12 h to ∼123 nm after 2 weeks, accompanied by a modest rise in the PDI (0.13–0.19) and a decline in the zeta potential (+12.1 to +9.1 mV), consistent with slow surface hydrolysis and limited aggregation under aqueous conditions, as previously reported for ZIF-8.^13,14,18^ PD@ZIF-8 showed greater dimensional changes (109–147 nm; PDI: 0.12–0.28), reflecting the influence of PD incorporation on framework stability. In contrast, the PDI fell below 0.5 for PD@ZIF-8/CS, showing a significantly larger but more controlled increase (214–259 nm), indicating good short-term dispersion stability that is likely due to the steric stabilization provided by the CS polymeric shell.^14,21,24^ Long-term sedimentation studies will be conducted in the future, as the zeta potential of PD@ZIF-8/CS (−3.88 mV at pH 7.4) is close to the limit of electrostatic stability for conventional particles. Increasing the temperature from 25 °C to 60 °C (thermal stability; Fig. 6b and e) induced progressive size growth for all samples, but the extent of destabilization differed. ZIF-8's particle size increased from 98 to 119 nm, while PD@ZIF-8 showed a size increase of 11 nm (108–119 nm) with PDI broadening at 60 °C.^13^ PD@ZIF-8/CS also showed an 18 nm size increase from 205 to 223 nm. However, the relative size change (as a percentage of the initial particle size) of PD@ZIF-8/CS was also smaller (8.8%) than that of PD@ZIF-8 (10.2%), again confirming the improved thermal colloidal stability of the CS coating.^14^ In addition, a slight reduction in the surface charge (−3.9 to −2.0 mV) confirmed that the polymer shell enhanced colloidal integrity under hyperthermic conditions.^14^ In terms of pH stability (Fig. 6c and f), both ZIF-8 and PD@ZIF-8 were relatively stable under neutral/basic conditions (pH 7.4–9), but showed an increased size and an altered zeta potential at acidic pH values, consistent with the well-known acid lability of ZIF-8 frameworks.^13,14^ Under acidic conditions, the dual effect of PD@ZIF-8/CS was demonstrated by (i) the red shift of the particle size from 215 to 242 nm and the broadening of the PDI and (ii) the shift of the zeta potential from negative (−3.9 mV at pH 7.4; Fig. 2d) to positive (3.18 at pH 5.0; Fig. 6f) and +5.9 mV at pH 4.0, which corresponds to the protonation of CS amino groups.^14^ Importantly, the near-neutral zeta potential at the physiological pH is also functionally advantageous since the protonation of the CS amino groups (–NH_2_ to –NH_3_^+^) occurs in the acidic infection microenvironment (pH 5.5–6.5), causing a shift to more positive potentials (+5.9 mV at pH 4; Fig. 6f) and thereby increasing the adhesion of bacterial membranes and accelerating the drug release rate.^14,28,54,55^ These DLS findings demonstrate that ZIF-8 and PD@ZIF-8 suffer from time-, temperature-, and pH-induced destabilization, while PD@ZIF-8/CS retains colloidal integrity and introduces pH-responsive behavior. The CS coating, therefore, not only improves aqueous stability but also provides a functional stimulus-responsive release mechanism, enhancing the potential of PD@ZIF-8/CS as a smart drug delivery platform.

### 3.3. Antibacterial study

**Fig. 7:**
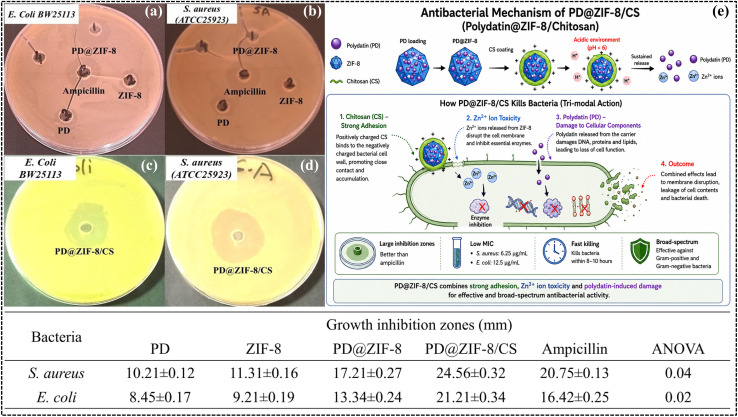
Antibacterial activity of PD, ZIF-8, PD@ZIF-8, PD@ZIF-8/CS, and ampicillin against *E. coli* and *S. aureus*: (a–d) inhibition zones and (e) proposed antibacterial mechanism.

The antibacterial efficacy of PD, ZIF-8, PD@ZIF-8, and PD@ZIF-8/CS was evaluated against *S*. *aureus* and *E*. *coli* using the agar well diffusion method (Fig. 7a–d). All materials exhibited clear inhibition zones, indicating effective bacterial growth suppression, although the extent of inhibition varied with the material composition. Pure PD displayed modest inhibition zones of 10.21 ± 0.12 mm against *S. aureus* and 8.45 ± 0.17 mm against *E. coli*, suggesting limited bacteriostatic potential in the free form. The antibacterial effect of PD is believed to be due to its polyphenolic stilbenoid structure, which is capable of disrupting the integrity of cell membranes by modifying the lipid permeability and protein conformation of bacteria.^56^ Furthermore, PD has been shown to inhibit the expression of key virulence regulatory proteins in MRSA, like caseinolytic protease P (ClpP), and to inhibit the activity of efflux pump (AcrAB-TolC), reducing bacterial persistence and resistance.^6^ Moreover, PD regulates intracellular oxidation and activates the Nrf-2/ARE antioxidant pathway, which is responsible for its overall antibacterial and cytoprotective effects.^6,56,57^

In comparison, ZIF-8 showed slightly improved inhibition zones (11.31 ± 0.16 mm for *S. aureus* and 9.21 ± 0.19 mm for *E. coli*) due to the slow release of Zn^2+^, which interferes with bacterial membrane integrity and enzymatic processes.^5^ When PD was encapsulated within ZIF-8 (PD@ZIF-8), the inhibition zones significantly increased to 17.21 ± 0.27 mm (*S. aureus*) and 13.34 ± 0.24 mm (*E. coli*), suggesting a synergistic antibacterial enhancement due to the combined action of redox-active PD and Zn^2+^ release.^5,6,56,57^ The most remarkable antibacterial performance was observed for PD@ZIF-8/CS, which exhibited inhibition zones of 24.56 ± 0.32 mm and 21.21 ± 0.34 mm against *S. aureus* and *E. coli*, respectively, values notably higher than those of the standard antibiotic ampicillin (20.75 ± 0.31 mm and 16.42 ± 0.25 mm, respectively). These values compare favorably with other ZIF-8-based DDS, *i.e.*, Mag@ZIF-8 (26.5/22.8 mm),^14^ Glab@ZIF-8 (26.2/23.8 mm),^13^ GPS@ZIF-8 (25/22 mm),^18^ ZIF-8@Rutin (20.1/20.1 mm),^34^ and PHY@ZIF-8 (20/18 mm),^15^ respectively, highlighting the excellent antibacterial efficacy of PD@ZIF-8/CS.

**Fig. 8:**
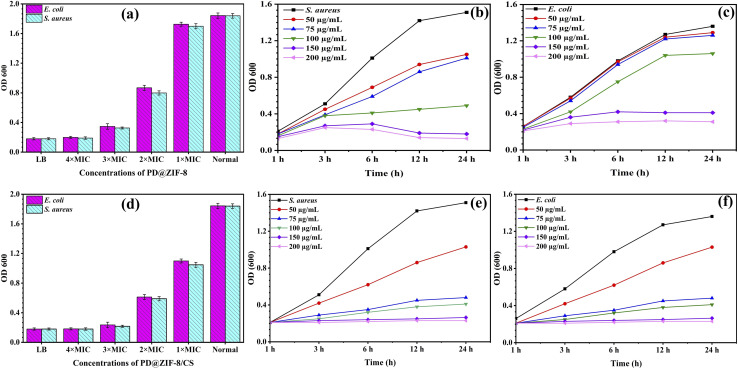
MIC of (a) PD@ZIF-8; time-kill assays against (b) *S. aureus* and (c) *E. coli*; MIC of (d) PD@ZIF-8/CS; and time-kill assays against (e) *S. aureus* and (f) *E. coli*.

The MIC values revealed a clear concentration-dependent reduction in bacterial growth. PD@ZIF-8 showed MIC values of 25 and 50 µg mL^−1^ (Fig. 8a), whereas PD@ZIF-8/CS achieved lower MICs of 6.25 µg mL^−1^ for *S. aureus* and 12.5 µg mL^−1^ for *E. coli* (Fig. 8d), respectively. These findings correlate well with the MIC of Mag@ZIF-8/CS (20 µg mL^−1^)^14^ and outperform the reported ZIF-8@curcumin (22 µg mL^−1^) and ZIF-8@CCM (22 µg mL^−1^).^28,58^

The improved MIC observed for PD@ZIF-8 and PD@ZIF-8/CS is attributed to their physicochemical properties, which include a high DLE of 91.34%, a particle size ranging from 108.6 to 205.8 nm, and pH-responsive switching of the surface charge in PD@ZIF-8/CS due to the CS coating, all of which facilitate bacterial adhesion, cellular uptake, and the prolonged corelease of PD and Zn^2+^ in acidic microenvironments.^59^

**Fig. 9:**
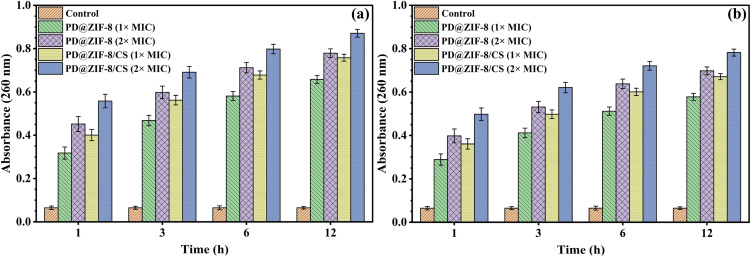
Absorbance values (260 nm) of intracellular contents released from (a) *S. aureus* and (b) *E. coli* treated with PD@ZIF-8 and PD@ZIF-8/CS at 1× and 2× MIC concentrations over 1–12 h.

Time-kill kinetics demonstrated that PD@ZIF-8/CS rapidly reduced viable bacterial counts within 4 h and achieved nearly complete eradication within 8 h for *S. aureus* and 10 h for *E. coli* (Fig. 8e and f). In contrast, PD@ZIF-8 required prolonged exposure (>12 h) for partial bacterial inhibition (Fig. 8b and c). Intracellular content leakage was also evaluated by measuring the absorbance at 260 nm after treatment with 1× and 2× MICs for 1, 3, 6 and 12 h to further confirm the damage to the membrane level. The untreated control had a minimal absorbance (∼0.065) throughout, indicating the stability of the membrane in the absence of treatment. Both formulations showed a time- and concentration-dependent rise in absorbance for both strains. Against *S. aureus*, PD@ZIF-8 at 1× and 2× MICs produced absorbance values reaching 0.658 ± 0.018 and 0.779 ± 0.020 within 12 h, respectively, while PD@ZIF-8/CS achieved markedly higher values of 0.758 ± 0.016 and 0.871 ± 0.018, respectively (Fig. 9a). Both formulations exhibited relatively low leakage against *E. coli*, with PD@ZIF-8/CS exhibiting 0.671 ± 0.014 and 0.782 ± 0.016 at the 1× and 2× MICs, respectively, after 12 h (Fig. 9b), which is related to the extra protection provided by the outer membrane of Gram-negative bacteria.^60^

The superior antibiotic activity of PD@ZIF-8/CS is explained by a synergistic tri-modal mechanism involving Zn^2+^ toxicity,^5,61^ the PD-induced damage of metabolic functions, and CS-induced membrane adhesion (Fig. 7e). Bacterial metabolic activity creates an acidic microenvironment (pH 5.5–6.5), which protonates 2-MIM linkers, leading to the partial disassembly of the ZIF-8 framework and the release of Zn^2+^ and PD.^1,48,62^ The released Zn^2+^ leads to the disruption of membrane integrity and the inhibition of important enzymatic activities,^5^ and the released PD, at the same time, damages DNA, proteins and lipids of the cell.^6,56,57^ Importantly, under the same acidic conditions, the surface charge of CS changes from −3.88 mV at pH 7.4 to +5.9 mV at pH 4 (Fig. 6f), leading to strong electrostatic binding with the negatively charged bacterial cell wall and localizing antibacterial agents at the infection site.^14,24,36^ This physical and chemical mechanism, in combination, leads to the enhanced inhibition zones, decreased MIC, accelerated time-kill curves and increased membrane leakage for PD@ZIF-8/CS, making it a powerful and versatile nanoplatform for advanced biomedical applications. The proposed mechanistic findings of this study are recommended to be further extended in future studies by performing CFU counts, live/dead bacterial staining, and direct measurements of ROS.

### 3.4. Drug release profiles of PD@ZIF-8 and PD@ZIF-8/CS and drug release kinetics

**Fig. 10:**
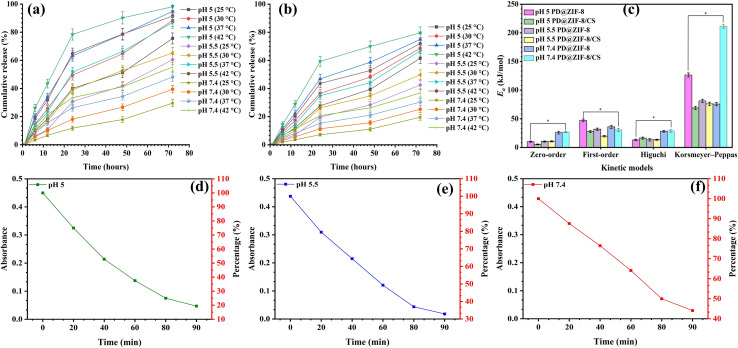
Release profile of PD from (a) PD@ZIF-8 and (b) PD@ZIF-8/CS; (c) release activation energies (*E*_a_) for PD@ZIF-8 and PD@ZIF-8/CS (* indicates significance determined by one-way ANOVA (*p* value < 0.05)); and stability of PD at (d) pH 5, (e) pH 5.5, and (f) pH 7.4.

The cumulative drug release profiles of PD@ZIF-8 (Fig. 10a) and PD@ZIF-8/CS (Fig. 10b) clearly demonstrated a strong pH and temperature dependence, reflecting the acid-labile nature of the ZIF-8 framework and the diffusion-barrier function of the CS coating. At pH 5.0 and 37 °C, PD@ZIF-8 exhibited a rapid release behavior (Fig. 10a), reaching nearly 95% release within 72 h, with the majority of release occuring within first 20 h, whereas at the physiological pH of 7.4, release was significantly suppressed, not exceeding ∼48% even after 72 h (*t*_0_ ≈ 72 h). This behavior is consistent with the established instability of ZIF-8 under acidic environments, where the protonation of 2-MIM linkers leads to Zn–N bond cleavage and framework disassembly, a process reported in several ZIF-8-based drug carriers.^5,13,15,18^ Such an accelerated degradation ensures efficient intracellular drug liberation in acidic endosomes or tumor tissues while minimizing premature leakage in systemic circulation.^63^ Upon CS coating (Fig. 10b), the release profiles were markedly altered. At pH 5.0 and 37 °C, PD@ZIF-8/CS released only ∼75% of the drug in 72 h with a *t*_0_ of ∼29 h, while at neutral pH, release was restricted to ∼30% over the same duration. The CS layer not only provided a diffusion barrier, slowing down the outward transport of the drug,^64^ but also stabilized the ZIF-8 core against premature collapse by shielding surface Zn^2+^ anodes from direct proton attack.^14,45^ As a result, the initial burst observed in ZIF-8 (∼34% at 12 h under acidic conditions, Fig. 10a) was significantly reduced to ∼22% in the CS-coated formulation (Fig. 10b). Similar effects have been observed in polymer-modified MOFs,^65,66^ where burst release is suppressed, but the quantitative delay in *t*_0_ and the pronounced pH-selectivity observed here indicate a more effective control.^14^ Moreover, the shift from a two-step release in PD@ZIF-8 to a more sustained and controlled release profile in PD@ZIF-8/CS is in agreement with the release profile of previously reported CS-coated MOFs.^14,36,64,67^ Hence, PD@ZIF-8/CS showed lower burst release, pH-responsive drug release, and negligible leakage in the physiological range, making it an ideal drug delivery system for antibacterial drugs.

Following drug release evaluation, we assessed the intrinsic pH stability of free PD to separate ZIF-assisted release from direct drug degradation at pH 7.4, 5.5, and 5.0 over 90 minutes (Fig. 10d–f). However, PD showed about 44.1% recovery after 90 minutes at the physiological pH of 7.4 (Fig. 10f), suggesting the relatively moderate stability of PD at neutral pH. The degradation rate was found to be progressive in acidic media, with ∼32.9% at pH 5.5 (Fig. 10e) and ∼19.4% at pH 5.0 (Fig. 10d), indicating significant degradation in acidic media. This is believed to be due to the acid-catalyzed hydrolysis of PD's C-glycosidic bond, which generates resveratrol, a bioactive aglycone with established antioxidant and anti-inflammatory activities.^6,8^ Due to this, acidic tumor and endosomal microenvironments release PD at the same time and convert part of it to resveratrol, which may have synergistic therapeutic effects.^68,69^ However, importantly, the observed free-PD degradation also highlights the necessity of a protective carrier system: unencapsulated PD is rapidly degraded at endosomal/lysosomal pH values (∼5.0), which would greatly decrease its bioavailability if it were not encapsulated. The framework dissolution of ZIF-8 is the main mechanism responsible for the release of the drug under acidic conditions, and the chitosan-coated outer layer of the nanostructure further protects the drug against premature degradation, thereby illustrating the benefit of CS-coated ZIF-8 for pH-responsive and efficient PD delivery to acidic microenvironments.^24,39^

The drug release kinetics of PD@ZIF-8 and PD@ZIF-8/CS were systematically fitted with zero-order, first-order, Higuchi, and Korsmeyer–Peppas models across different pH and temperature conditions, followed by Arrhenius analysis to extract activation energies (*E*_a_).^13,14,70,71^ This integrated approach enabled us to distinguish not only the pathways of release but also the energetic and thermal requirements of each pathway. A comparative statistical study of all four models showed that the Korsmeyer–Peppas model exhibited the highest *R*^2^ values (0.978–0.995) compared to the zero-order (0.890–0.985), first-order (0.946–0.989) and Higuchi (0.946–0.991) models under all tested pH and temperature conditions (Table S1). For PD@ZIF-8 (Table S1), drug release under acidic conditions (pH 5.0 and 5.5) was best explained by the Higuchi and Korsmeyer–Peppas models. At pH 5.0 and 25 °C, Higuchi fitting yielded *R*^2^ = 0.981 (*k* = 9.99), while Peppas fitting provided *R*^2^ = 0.978 with *k* = 0.026 and *n* = 0.793, demonstrating inconsistent non-Fickian transport, reflecting combined diffusion and ZIF-8 framework relaxation, which could be attributed to a mechanistic distinction not captured by the zero-order, first-order, or Higuchi models. With increasing temperature (30–42 °C), *R*^2^ values remained high (>0.95) and rate constants increased (Higuchi *k* increased from 9.99 at 25 °C to 14.2 at 42 °C), confirming that higher thermal energy facilitated faster diffusion.^13^ Correspondingly, *E*_a_ values for Higuchi release were low (13.08–13.45 kJ mol^−1^ across acidic pH), highlighting diffusion-driven release, whereas Peppas *E*_a_ was much higher (126.5 kJ mol^−1^ at pH 5.0; 81.2 kJ mol^−1^ at pH 5.5; Fig. 10c and Table S2), reflecting additional energy barriers for matrix relaxation.^72^ For PD@ZIF-8/CS at acidic pH, a similar but more pH-responsive behavior was observed. At pH 5.0, Higuchi fitting at 25 °C gave *R*^2^ = 0.965 (*k* = 7.73), while Peppas fitting yielded *R*^2^ = 0.980 (*k* = 0.014, *n* = 0.879). Notably, the Peppas exponent, *n*, increased with the temperature, reaching >1.0 at 42 °C, consistent with a super case-II mechanism, where polymer relaxation and erosion dominate.^14^ Activation energies further support this interpretation: zero-order *E*_a_ dropped to 5.26 kJ mol^−1^ at pH 5 compared to 10.02 kJ mol^−1^ for bare ZIF-8 (Fig. 10c and Table S2), confirming that CS protonation facilitates thermally assisted hydration and diffusion.^14^

At the physiological pH (7.4), PD@ZIF-8 followed a markedly different pattern. As seen in Table S1, the first-order fit had *R*^2^ = 0.984 at 25 °C (*k* = 0.005), *R*^2^ = 0.989 at 30 °C (*k* = 0.007), and *R*^2^ = 0.974 at 37 °C (*k* = 0.009). This shows the fact that the ZIF-8 mechanism remains intact at pH 7.4 and that the mechanisms are purely first-order.^13,14,18^ Increasing the temperature accelerated release (*k* nearly doubling between 25 °C and 42 °C), but *E*_a_ values remained moderate: 35.9 kJ mol^−1^ for the first-order fit, compared to 25.9–28.2 kJ mol^−1^ (Fig. 10c and Table S2) for zero-order and Higuchi fits, indicating that neutral pH imposes stronger barriers to diffusion relative to acidic conditions. For PD@ZIF-8/CS at pH 7.4, thermal effects were even more striking. While first-order fits remained excellent (*R*^2^ ≈ 0.98, *k* = 0.003–0.006 across 25–42 °C), Peppas fitting exhibited the strongest agreement at higher temperatures (*R*^2^ = 0.992, *k* = 0.004, *n* ≈ 0.99). However, the corresponding *E*_a_ for Peppas release reached an exceptionally high value of 211.6 kJ mol^−1^, compared with only 75.7 kJ mol^−1^ for PD@ZIF-8 (Fig. 10c and Tables S1, S2). This suggests that the CS shell imposes a thermally sensitive diffusion barrier under neutral conditions, delaying drug escape until sufficient heating and hydration enable polymer relaxation. Such a behavior is consistent with previous reports of CS coatings acting as “gatekeepers” in polymer–MOF hybrids.^14^

**Fig. 11:**
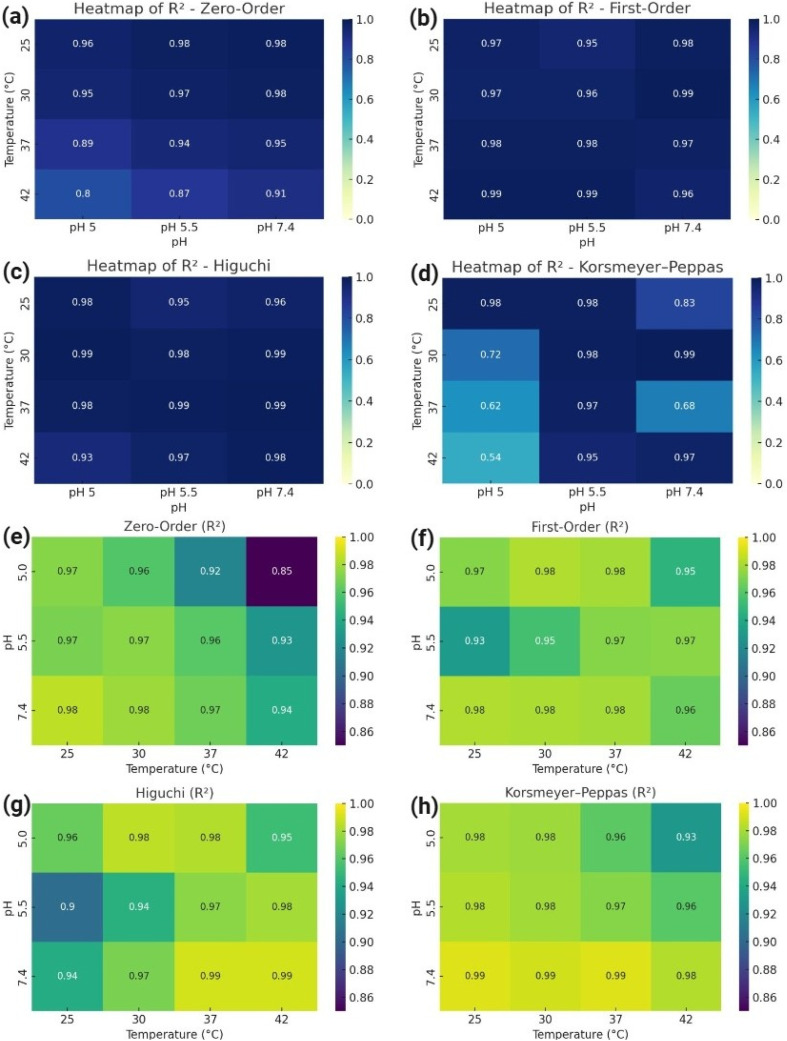
Heatmaps illustrating the *R*^2^ values of four kinetic models for polydatin release from (a–d) PD@ZIF-8 and (e–h) PD@ZIF-8/CS under varying pH and temperature conditions.

The combined model fitting (Table S1), heatmaps (Fig. 11), radar plots (Fig. S7 and S8) and *E*_a_ analysis (Fig. 10c) demonstrate that CS has a dual role: it decreases the activation energy and boosts the release rate under acidic microenvironmental conditions (*E*_a_ decreases to as low as 5.26 kJ mol^−1^) while significantly increasing the activation energy under physiological pH conditions (*E*_a_ increases up to 211.6 kJ mol^−1^). Both the Peppas exponent, *n*, and the experimental data clearly delineate the mechanism of transport under acidic conditions: under acidic conditions, the exponent *n* = 0.793–0.990 is indicative of anomalous non-Fickian transport behavior due to both the diffusion and relaxation of the ZIF-8 framework, whereas *n* > 1.0 for PD@ZIF-8/CS at 42 °C suggests that the super case-II transport behavior is dominated by the swelling and erosion of the CS polymer.^14,70,71^ This systematic model selection and mechanistic interpretation make the Korsmeyer–Peppas model the most informative kinetic descriptor for pH- and temperature-responsive ZIF-8/CS drug delivery systems. This thermal and pH dual-responsiveness underscores the novelty of PD@ZIF-8/CS, offering a highly selective drug delivery platform with minimal leakage in circulation and enhanced release in tumor-relevant acidic, hyperthermic niches. Overall, the combined kinetic modeling and Arrhenius analysis demonstrate that PD@ZIF-8 release is primarily diffusion-driven at acidic pH and concentration-dependent at physiological pH, whereas PD@ZIF-8/CS exhibits a unique dual responsiveness. The CS shell markedly lowers *E*_a_ under acidic conditions (5.26 kJ mol^−1^*vs.* 10.02 kJ mol^−1^ for ZIF-8) but raises it to exceptionally high levels at neutral pH (211.6 kJ mol^−1^*vs.* 75.7 kJ mol^−1^ for ZIF-8), imparting precise control over release. This pH- and temperature-sensitive gating effect highlights PD@ZIF-8/CS as a novel and highly promising drug carrier with potential for tumor-targeted delivery.

## 4. Conclusion

This study demonstrates an efficient one-pot room-temperature synthesis route for developing PD-loaded ZIF-8 (PD@ZIF-8) and its CS-coated derivative (PD@ZIF-8/CS), introducing a distinctive hybrid drug delivery platform that merges natural bioactivity with MOF versatility. Quantitative characterization confirms that PD incorporation and CS coating markedly influence the structural order and functionality. XRD analysis shows that the crystallinity of ZIF-8 (68.56%) increases to 72.61% for PD@ZIF-8 and 75.90% for PD@ZIF-8/CS, while the crystallite size decreases from 42.3 to 31.8 and 25.9 nm, respectively, indicating enhanced nucleation and lattice compactness. SEM and DLS analyses reveal a stepwise size evolution from 85.2 to 115.4 and 294.8 nm, validating successful PD loading and complete CS encapsulation. Further, PD@ZIF-8 demonstrates a high drug-loading efficiency of 91.34% and exhibits a strong pH-dependent release, achieving 92.5% release at pH 5.0 and 37 °C within 72 h. The CS coating decreases the burst release by ∼35% and prolongs the *t*_50_ from 18.4 h to 38.6 h, following the Korsmeyer–Peppas kinetic model. The antibacterial activity of PD@ZIF-8/CS is significantly enhanced, with inhibition zones of 24.6 ± 0.4 mm for *S. aureus* and 21.2 ± 0.3 mm for *E. coli* and an MIC of 6.25 µg mL^−1^, confirming a strong synergistic mechanism among PD, Zn^2+^ release, and CS adhesion. This work uniquely demonstrates that one-pot PD encapsulation coupled with CS surface engineering yields a biocompatible, structurally reinforced, and pH-sensitive nanoplatform with dual functionality, sustained release and potent antibacterial action, representing a promising candidate for further biological evaluation toward antibacterial drug delivery applications.

## Conflicts of interest

The authors declare that they have no known competing financial interests or personal relationships that could have appeared to influence the work reported in this paper.

## Supplementary Material

RA-OLF-D6RA04037C-s001

## Data Availability

The data supporting the findings of this study are available from the corresponding author upon reasonable request. Supplementary information (SI) is available. See DOI: https://doi.org/10.1039/d6ra04037c.
