# Supplementary material for: Biocompatible chitosan-coated polydatin@ZIF-8 MOF nanostructures: synthesis, drug delivery and enhanced antibacterial properties
Source: RSC Adv. 2026 Aug 11;16(40):46808–26. doi: 10.1039/d6ra04037c (PMC13459614; doi:10.1039/d6ra04037c)
Supplement: Supplementary file 1 [file RA-016-D6RA04037C-s001.pdf]

Supplementary Information (SI) for RSC Advances.
This journal is © The Royal Society of Chemistry 2026

Biocompatible Chitosan-Coated Polydatin@ZIF-8 MOF Nanostructures: Synthesis, Drug delivery and Enhanced Antibacterial Properties

Abdul Ghaffar[1], Safdar Ali Amur[2], Quratulain Khuhro[3], Najaf Ali Soomro[3], Mujahid Mehid[4], Jun Liu[1*], Khalid Hussain Thebo[5*], Muhammad Atiq Ur Rehman[6], Akbar Ali[7], Ahmed Nadeem[8]

[1]*Key Laboratory of Air-Driven Equipment Technology of Zhejiang Province, College of Mechanical Engineering, Quzhou University, Quzhou 324000, Zhejiang, China.*

[2]*Department of Chemistry, Faculty of Natural Science, Government College University, Hyderabad Sindh, Pakistan, 71000.*

[3]*Institute of Biochemistry, University of Sindh, Jamshoro, 76080, Pakistan.*

[4]*Hangzhou International Innovation Institute, Beihang University, China.*

[5]*Department of Chemistry, Mirpur University of Science & Technology (MUST), A&J Kashmir, Pakistan.*

[6]*Department of Materials Science and Engineering, Institute of Space Technology Islamabad, 4400, Pakistan.*

[7]*MIIT Key Laboratory of Critical Materials Technology for New Energy Conversion and Storage, State Key Laboratory of Urban Water Resource and Environment, School of Chemistry and Chemical Engineering, Harbin Institute of Technology, Harbin 150001, PR China.*

[7]*Department of Pharmacology and Toxicology, College of Pharmacy, King Saud University, Riyadh 11451, Saudi Arabia.*

Corresponding author Email: 36119@qzc.edu.cn (J.L.)[*]
thebokhalid@gmail.com (K.H.T)[*]

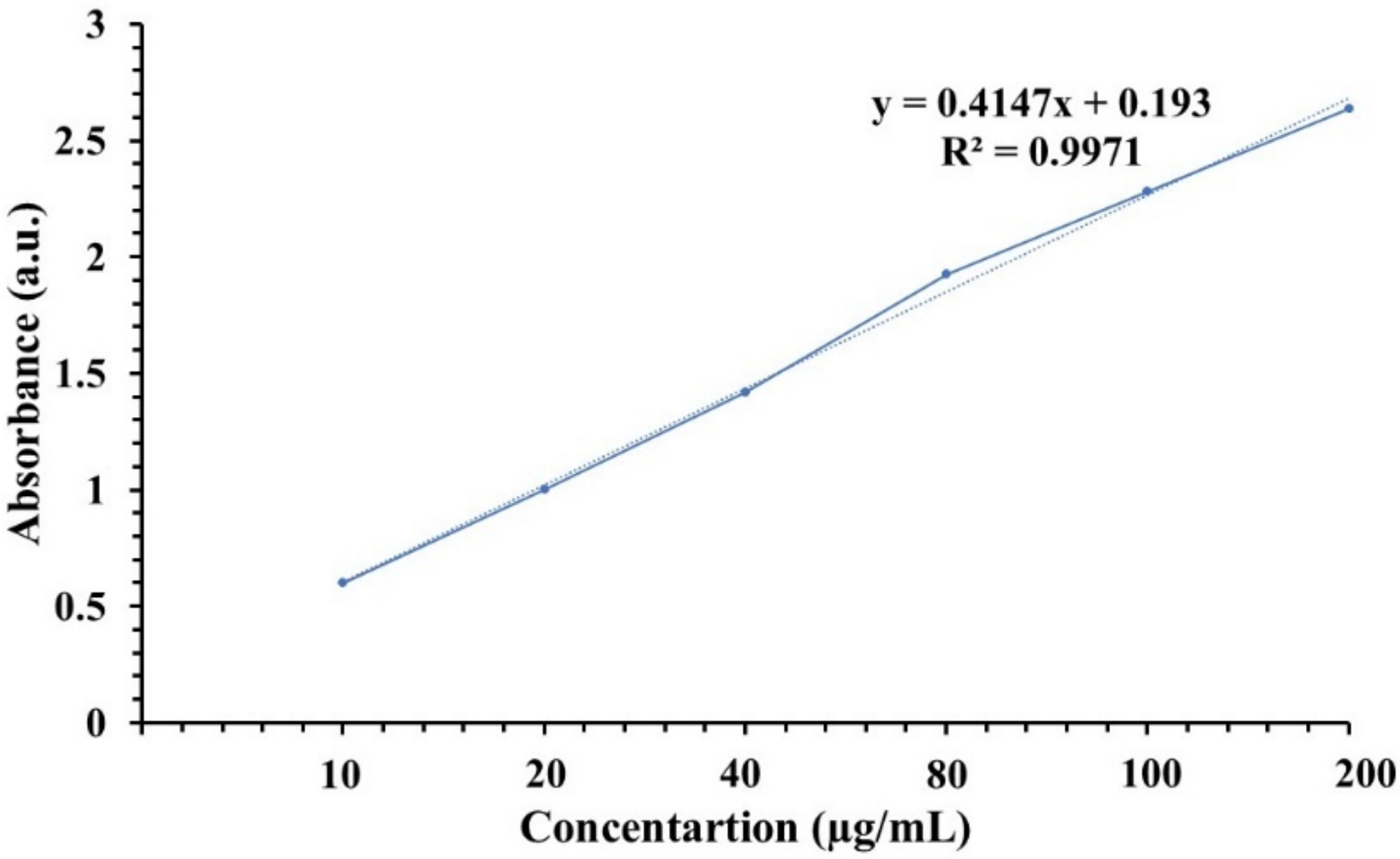

Figure S1 Calibration curve of Polydatin (PD): Shows a linear correlation between absorbance and concentration (10–200 µg/mL) at λ_max = 313 nm. The regression equation ($y = 0.4147x + 0.193$, $R^2 = 0.9971$) demonstrates excellent linearity in accordance with the Beer–Lambert law, confirming the reliability of the UV–Vis quantification method.

Drug Release Model Equations and Theoretical Background

Zero-order model:

Describes a system where the release rate is constant and independent of drug concentration. This is typical of controlled-release matrices. A linear plot of cumulative drug released (Q_t) versus time (t) indicates zero-order kinetics.

Equation: $Q_t = Q_0 + k_0 t$

where Q_t is the amount of drug released at time t, Q_0 is the initial amount, and k_0 is the zero-order release constant.

First-order model:

Assumes the release rate is concentration-dependent, often applied to dissolution-controlled systems. A linear relationship between log($Q_0 - Q_t$) and t indicates first-order kinetics.

Equation: $\log Q_t = \log Q_0 - \frac{k_1 t}{2.303}$

where Q_0 is the initial amount of drug, Q_t is the amount released at time t and k_1 is the first-order release constant.

Higuchi model:

Based on Fick's law of diffusion, this model describes drug release from a homogeneous matrix system where the release is proportional to the square root of time.

Equation: $Q_t = k_H \sqrt{t}$

where k_H is the Higuchi dissolution constant.

Korsmeyer–Peppas model:

A semi-empirical model describing both Fickian and non-Fickian release from polymeric matrices. The release exponent (n) characterizes the mechanism: Fickian diffusion ($n \leq 0.5$), anomalous transport ($0.5 < n < 1$), or super case-II transport ($n \geq 1$).

This semi-empirical model is used to describe both Fickian and non-Fickian release behaviors, especially in polymeric systems. The release exponent 'n' characterizes the mechanism: Fickian diffusion ($n \leq 0.5$), non-Fickian ($0.5 < n < 1$), and super case-II transport ($n \geq 1$).

Equation: $$Korsmeyer\ Peppas \frac{M_t}{M_\infty} = k_{KP} t^n$$

where M_t/M_∞ is the fraction of drug released at time t, k_{KP} is the release rate constant, and n is the release exponent.

Arrhenius analysis

To assess the temperature dependence of release kinetics, Arrhenius analysis was applied to the rate constants (k) obtained from each model. Temperatures were converted to Kelvin (T), and ln k was plotted against the reciprocal of absolute temperature (1/T). Linear regression was performed according to the Arrhenius equation:

$$\text{In}\, k = \text{In}\, A - \frac{E_a 1}{R\, T}$$

where A is the pre-exponential factor, E_a is the activation energy, and R is the universal gas constant (8.314 $J{\cdot}mol^{-1}{\cdot}K^{-1}$). The slope of the regression line provided the activation energy as:

$$E_a = - slope \times R$$

Arrhenius plots (**Figure S2 and S3**) were generated for each kinetic model and pH condition, and the calculated E_a values (**Table S2**) were compared to elucidate the energy barrier and release mechanism under varying physiological environments. All calculations and graphical analyses were performed using Microsoft Excel 2016.

Table S1 Kinetic model fitting parameters of PD release from PD@ZIF-8 and PD@ZIF-8/CS at different pH and temperatures

Material	pH	Temp (°C)	Model	R^2	k value	n value
PD@ZIF-8	5	25	Zero-order	0.959	1.005	–
			First-order	0.971	0.019	–
			Higuchi	0.981	9.996	–
			Korsmeyer–Peppas	0.978	0.026	0.793
	5.5	25	Zero-order	0.978	0.975	–
			First-order	0.946	0.017	–
			Higuchi	0.946	9.427	–
			Korsmeyer–Peppas	0.978	0.010	0.990
	7.4	25	Zero-order	0.985	0.390	–
			First-order	0.984	0.005	–
			Higuchi	0.962	3.792	–
			Korsmeyer–Peppas	0.832	0.008	0.832
	5	30	Zero-order	0.947	1.168	–
			First-order	0.967	0.028	–
			Higuchi	0.991	11.75	–
			Korsmeyer–Peppas	0.717	0.043	0.717
	5.5	30	Zero-order	0.972	1.072	–
			First-order	0.965	0.021	–
			Higuchi	0.977	10.56	–
			Korsmeyer–Peppas	0.982	0.021	0.858
	7.4	30	Zero-order	0.979	0.523	–
			First-order	0.989	0.007	–
			Higuchi	0.986	5.157	–
			Korsmeyer–Peppas	0.995	0.015	0.760
	5	37	Zero-order	0.890	1.242	–
			First-order	0.978	0.039	–
			Higuchi	0.980	12.82	–
			Korsmeyer–Peppas	0.623	0.072	0.623
	5.5	37	Zero-order	0.936	1.171	–
			First-order	0.980	0.027	–
			Higuchi	0.986	11.82	–
			Korsmeyer–Peppas	0.973	0.038	0.750
	7.4	37	Zero-order	0.946	0.624	–
			First-order	0.974	0.009	–
			Higuchi	0.992	6.278	–
			Korsmeyer–Peppas	0.675	0.027	0.605
	5	42	Zero-order	0.797	1.262	–
			First-order	0.988	0.054	–
			Higuchi	0.933	13.43	–
			Korsmeyer–Peppas	0.540	0.540	0.111
	5.5	42	Zero-order	0.869	1.221	–
			First-order	0.986	0.034	–
			Higuchi	0.967	12.67	–
			Korsmeyer–Peppas	0.952	0.064	0.652
	7.4	42	Zero-order	0.909	0.699	–
			First-order	0.958	0.010	–
			Higuchi	0.985	7.155	–
			Korsmeyer–Peppas	0.968	0.043	0.605
**Material**	**pH**	**Temp (°C)**	**Model**	**R^2**	**k value**	**n value**
PD@ZIF-8/CS	5	25	Zero-order	0.973	0.973	–

		First-order	0.973	0.012	–
		Higuchi	0.965	7.736	–
		Korsmeyer–Peppas	0.980	0.014	0.879
5.5	25	Zero-order	0.970	0.764	–
		First-order	0.932	0.011	–
		Higuchi	0.903	7.254	–
		Korsmeyer–Peppas	0.983	0.005	1.076
7.4	25	Zero-order	0.985	0.260	–
		First-order	0.982	0.003	–
		Higuchi	0.942	2.501	–
		Korsmeyer–Peppas	0.992	0.004	0.992
5	30	Zero-order	0.959	0.921	–
		First-order	0.982	0.016	–
		Higuchi	0.985	9.181	–
		Korsmeyer–Peppas	0.978	0.023	0.807
5.5	30	Zero-order	0.974	0.817	–
		First-order	0.955	0.012	–
		Higuchi	0.945	7.917	–
		Korsmeyer–Peppas	0.979	0.010	0.966
7.4	30	Zero-order	0.978	0.333	–
		First-order	0.981	0.004	–
		Higuchi	0.970	3.262	–
		Korsmeyer–Peppas	0.989	0.008	0.808
5	37	Zero-order	0.918	1.009	–
		First-order	0.980	0.019	–
		Higuchi	0.983	10.26	–
		Korsmeyer–Peppas	0.962	0.071	0.748
5.5	37	Zero-order	0.961	0.898	–
		First-order	0.973	0.015	–
		Higuchi	0.974	8.891	–
		Korsmeyer–Peppas	0.973	0.018	0.862
7.4	37	Zero-order	0.967	0.400	–
		First-order	0.980	0.005	–
		Higuchi	0.990	3.978	–
		Korsmeyer–Peppas	0.992	0.014	0.723
5	42	Zero-order	0.846	1.073	–
		First-order	0.947	0.022	–
		Higuchi	0.953	11.19	–
		Korsmeyer–Peppas	0.930	0.050	0.688
5.5	42	Zero-order	0.928	0.962	–
		First-order	0.974	0.017	–
		Higuchi	0.980	9.722	–
		Korsmeyer–Peppas	0.961	0.029	0.772
7.4	42	Zero-order	0.942	0.477	–
		First-order	0.965	0.006	–
		Higuchi	0.990	4.805	–
		Korsmeyer–Peppas	0.983	0.673	0.021

k value units: Zero-order = $\%\cdot h^{-1}$, First-order = h^{-1}, Higuchi = $\%\cdot h^{-1/2}$, Korsmeyer–Peppas = $time^{-n}$

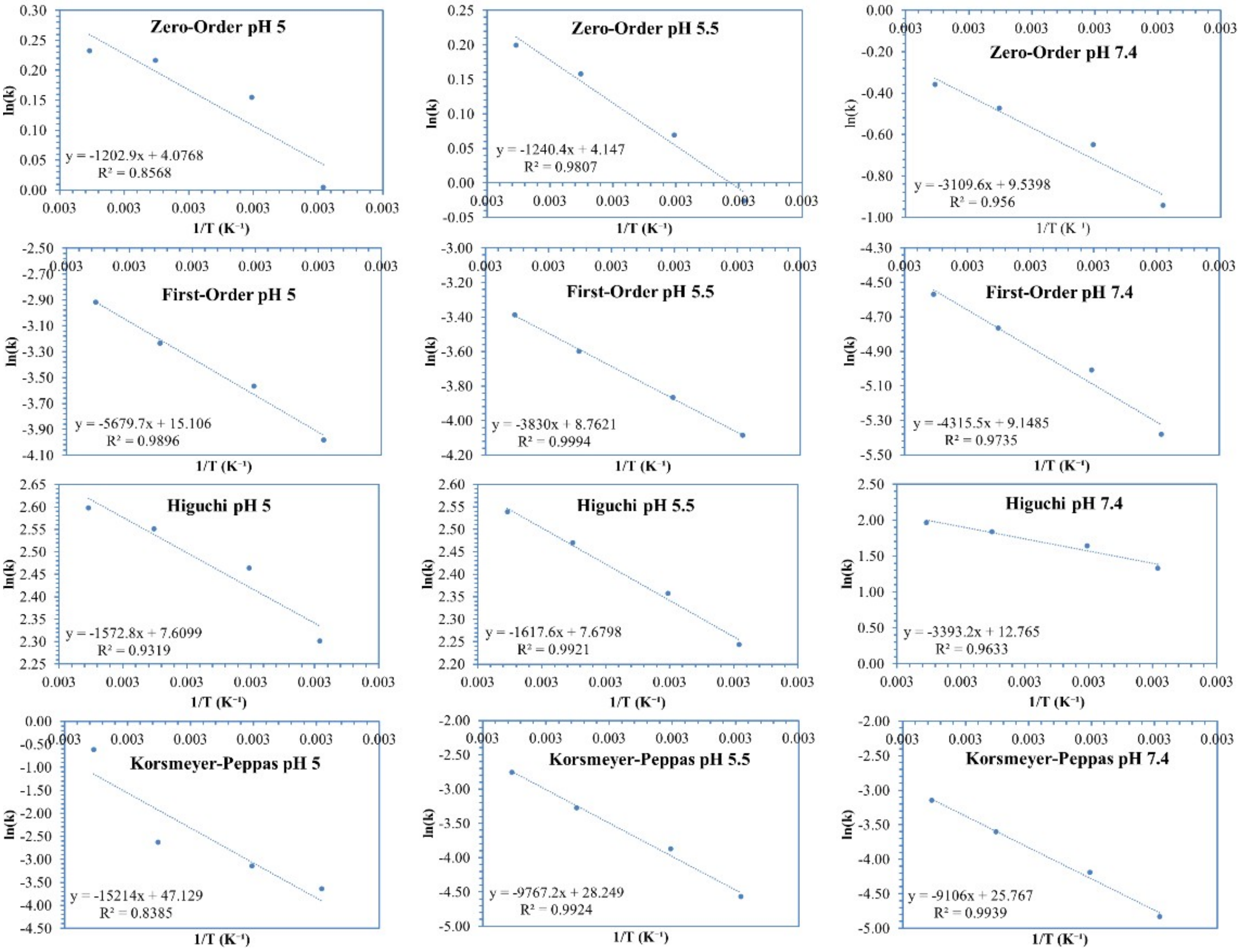

Figure 2 Arrhenius plots (*ln k* vs. *1/T*) for Zero-order, First-order, Higuchi, and Korsmeyer–Peppas kinetic models describing PD release from PD@ZIF-8 at pH 5.0, 5.5, and 7.4. Regression equations and R^2 values are shown for each fit: The linear regression fits (*ln k* vs. *1/T*) confirm good correlations (R^2 mostly >0.93), indicating reliable activation energy estimation. Among the models, the First-order and Korsmeyer–Peppas fits generally exhibited the strongest linearity (R^2 up to 0.999), suggesting that PD release is strongly governed by diffusion and degradation kinetics under different pH conditions.

Table S2 Activation energy (E_a) of drug release calculated from Arrhenius analysis

Model	Materials	E_a at pH 5	E_a at pH 5.5	E_a at pH 7.4

		(kJ/mol)	(kJ/mol)	(kJ/mol)
Zero-order	PD@ZIF-8	10.02	10.31	25.85
	PD@ZIF-8/CS	5.26	10.57	26.81
First-order	PD@ZIF-8	47.22	31.84	35.88
	PD@ZIF-8/CS	27.57	19.74	30.67
Higuchi	PD@ZIF-8	13.08	13.45	28.21
	PD@ZIF-8/CS	16.34	13.38	28.90
Korsmeyer–Peppas	PD@ZIF-8	126.5	81.20	75.71
	PD@ZIF-8/CS	68.9	76.03	211.57

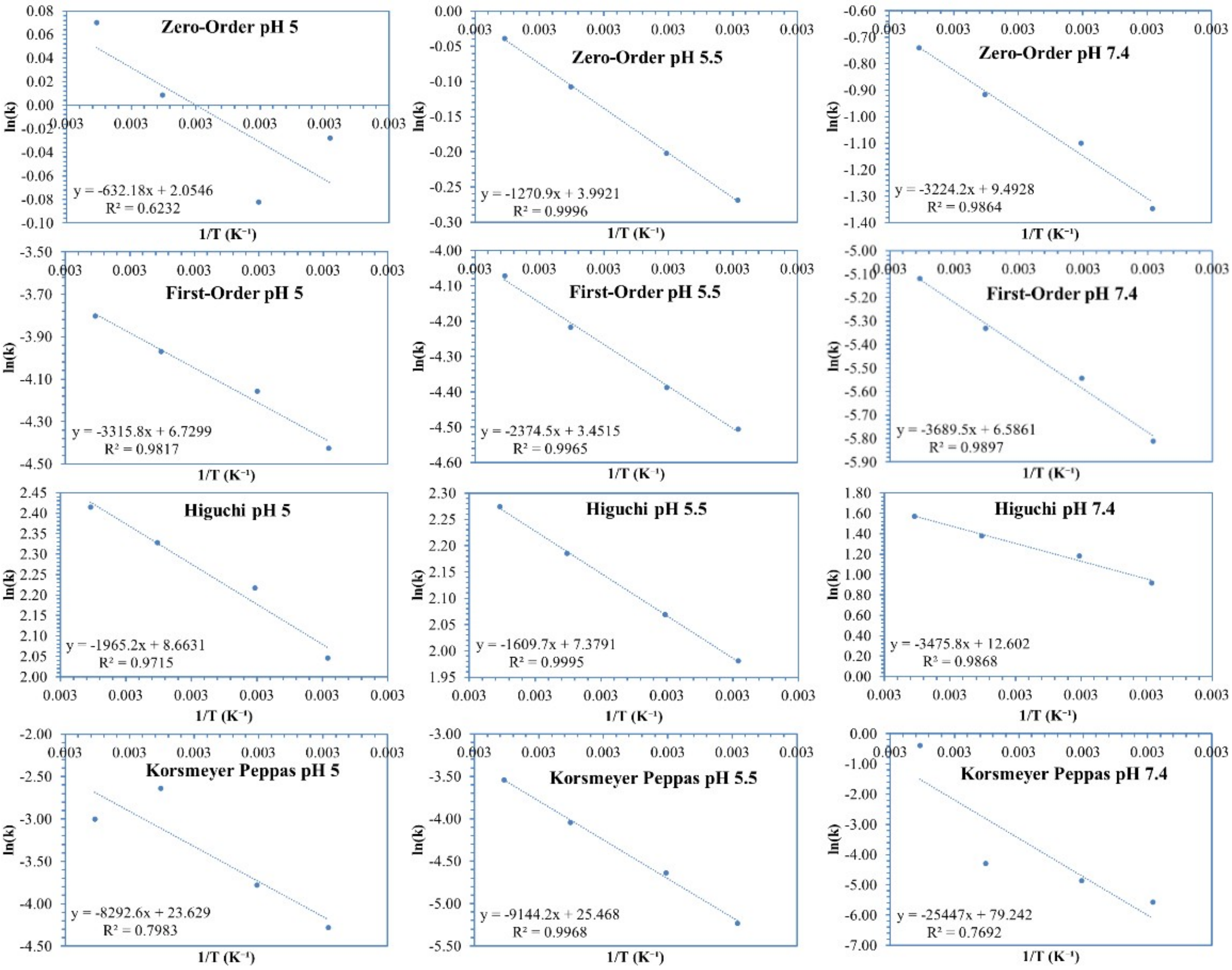

Figure 3 Arrhenius plots (*ln k* vs. *1/T*) for Zero-order, First-order, Higuchi, and Korsmeyer–Peppas models describing PD release from PD@ZIF-8/CS at pH 5.0, 5.5, and 7.4. Regression equations and R^2 values are provided for each fit: The linearity (R^2 mostly >0.97) indicates reliable kinetic–thermodynamic correlation. Compared to PD@ZIF-8, the ZIF-8/CS composite generally shows improved linearity in First-order and Higuchi models, highlighting a stronger diffusion-controlled release behavior. The slopes confirm model-dependent variations in activation energy, with diffusion-related models (Higuchi and Korsmeyer–Peppas) being more consistent across pH conditions.

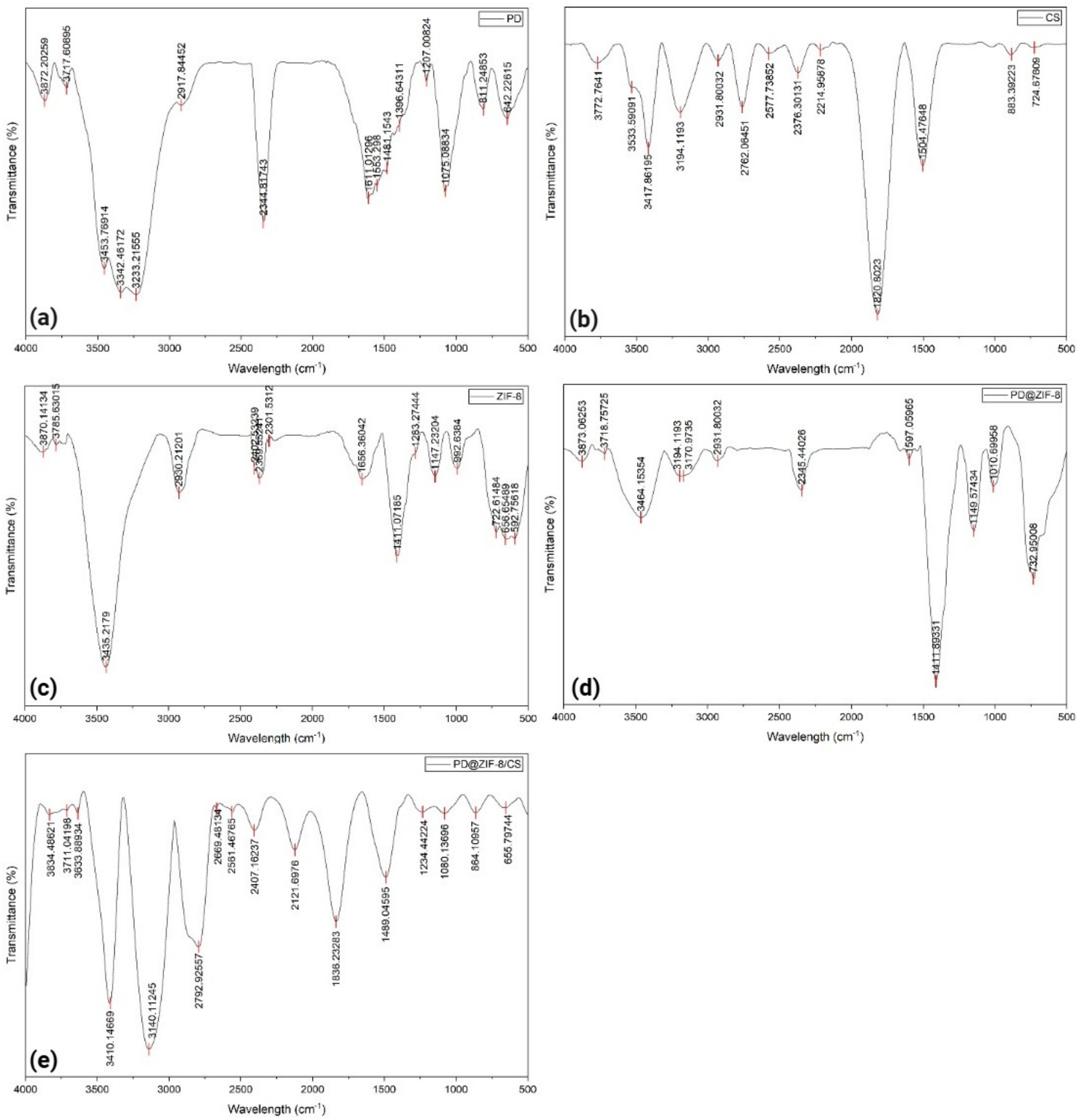

Figure S4 FTIR spectra of (a) PD, (b) CS, (c) ZIF-8, (d) PD@ZIF-8, and (e) PD@ZIF-8/CS: The spectra confirm the characteristic functional groups of each component, successful encapsulation of PD within ZIF-8, and subsequent CS coating to form the green-synthesized PD@ZIF-8/CS drug delivery system.

Table S3 FTIR spectral assignments of PD, CS, ZIF-8, PD@ZIF-8, and PD@ZIF-8/CS, showing characteristic peaks, and corresponding functional groups

Material	Observed Peaks (cm^{-1})	Assignment
**PD**	3365	O–H stretching (phenolic/alcoholic groups)
	2931, 2844	C–H stretching (aliphatic)
	1644	C=O stretching (ester), conjugated C=C
	1604, 1510, 1450	Aromatic skeletal C=C vibrations
	1274	C–O–C stretching (glycosidic linkage)
	1147, 1084	C–O stretching, aromatic ethers
	1028	C–O stretching (glucose moiety)
	996	Glycosidic bond vibration
	850–650	Aromatic C–H out-of-plane bending
**CS**	3435	O–H/N–H stretching (hydrogen bonding)
	2923, 2878	C–H stretching (aliphatic)
	1656	Amide I (C=O stretching)
	1590	Amide II (N–H bending, C–N stretching)
	1420–1375	CH_2 bending, C–N stretching
	1150, 1084, 1028	C–O–C and C–O stretching (glycosidic bonds)
	800–650	Saccharide ring skeletal vibrations
**ZIF-8**	3134, 2929, 2851	Aromatic/aliphatic C–H stretching
	1584	C=N stretching (imidazole ring)
	1450–1380	C–N stretching, imidazole ring deformation
	1274, 1147, 1071, 1042	In-plane imidazole bending, C–N stretching
	995–920	Ring deformation modes
	800–650	Aromatic C–H out-of-plane bending
	420	Zn–N stretching (framework fingerprint)
**PD@ZIF-8**	3370–3320	O–H stretching (PD, shifted due to interactions)
	2931, 2844	C–H stretching (PD + framework)
	1644	C=O stretching (PD), overlapped with ZIF-8
	1584	C=N stretching (ZIF-8 imidazole)
	1200–1000 (1147, 1084, 1028)	PD glycosidic C–O overlaps with imidazole C–N
	420	Zn–N stretching (framework intact)
**PD@ZIF-8/CS**	3435–3300	Broad O–H/N–H stretching (CS + PD + hydrogen bonding)
	2931, 2878	C–H stretching
	1650	Amide I (CS), overlapped with PD C=O
	1590	Amide II (CS) + ZIF-8 imidazole C=N
	1084, 1028	Glycosidic C–O–C (CS) + PD glucose moiety
	996	PD glycosidic vibration (reduced intensity)
	420	Zn–N stretching (framework intact after coating)

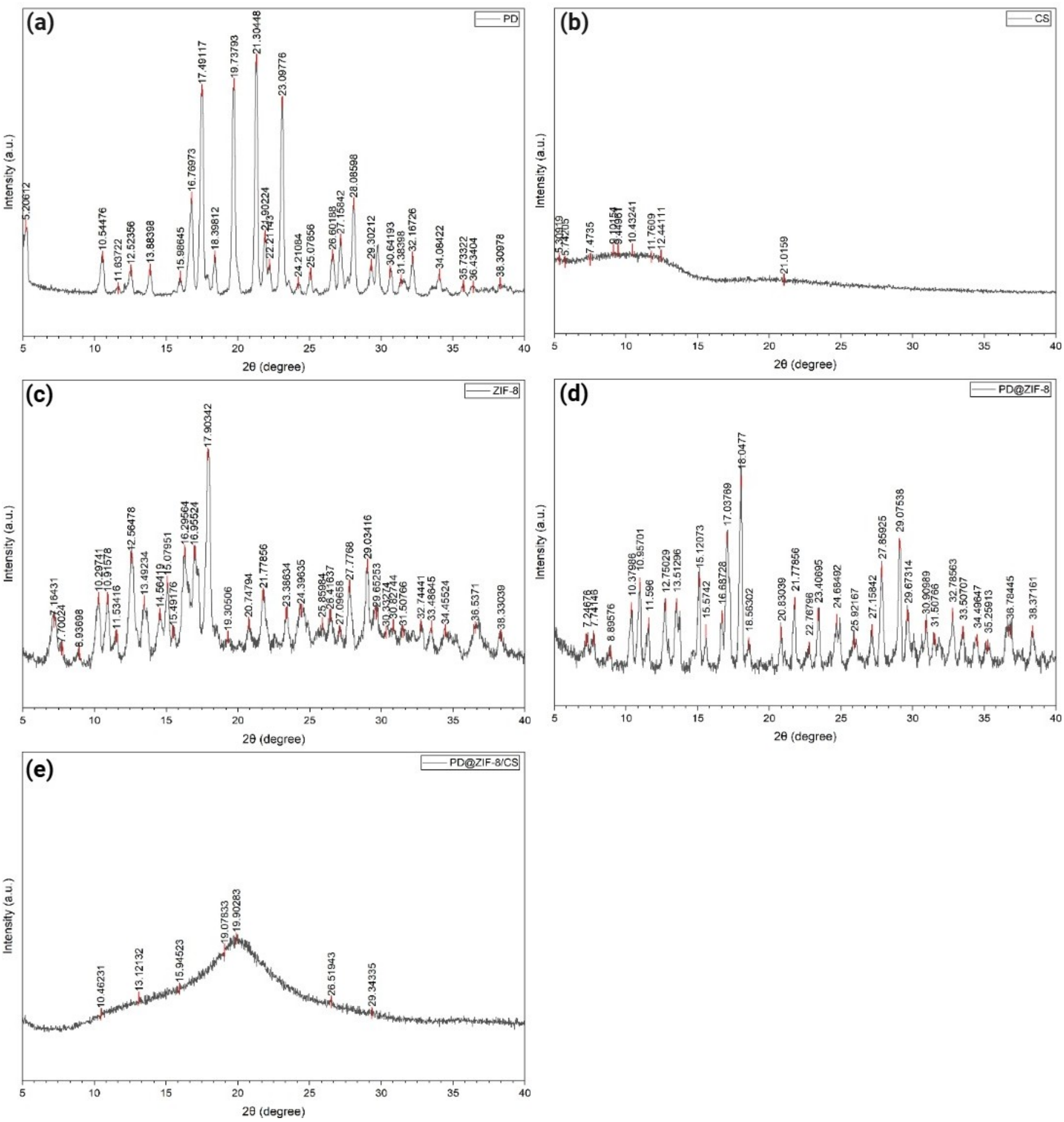

Figure 5 X-ray diffraction (XRD) patterns of (a) PD, (b) CS, (c) ZIF-8, (d) PD@ZIF-8, and (e) PD@ZIF-8/CS composites. The sharp peaks in PD confirm its crystalline nature, while the broad halo in CS reflects its semi-crystalline structure. ZIF-8 shows characteristic reflections of the sodalite-type framework. The preservation of major ZIF-8 peaks with reduced intensity in PD@ZIF-8 and PD@ZIF-8/CS indicates successful drug encapsulation and CS coating without loss of framework crystallinity.

Table S4 Summary of Quantitative XRD Parameters

Sample	Major Peak (2θ)	Crystallite Size (nm)	Crystallinity (%)	Structural Nature
**PD**	20.97°	37.6	66.98	Highly crystalline organic compound
**CS**	19.84°	28.4	77.57	Semi-crystalline polymer
**ZIF-8**	7.3°	42.3	68.56	Cubic sodalite-type framework
**PD@ZIF-8**	7.3°	31.8	72.61	Partial reduction in size and intensity
**PD@ZIF-8/CS**	7.3° + 20°	25.9	75.90	Reduced crystallinity due to polymer coating

Table S4 summarizes the quantitative XRD parameters of the individual components and composite materials. The pristine PD exhibited a sharp diffraction peak at 20.97°, confirming its highly crystalline organic structure, while CS showed a characteristic peak at 19.84°, indicating its semi-crystalline polymeric nature. The ZIF-8 sample displayed a distinct reflection at 7.3°, corresponding to its cubic sodalite-type framework. Upon PD incorporation (PD@ZIF-8), the crystallite size and peak intensity decreased slightly, suggesting partial structural modification. In PD@ZIF-8/CS, additional peaks around 20° appeared, reflecting the presence of the polymer coating and a reduction in overall crystallinity, which implies successful surface interaction between ZIF-8 and CS.

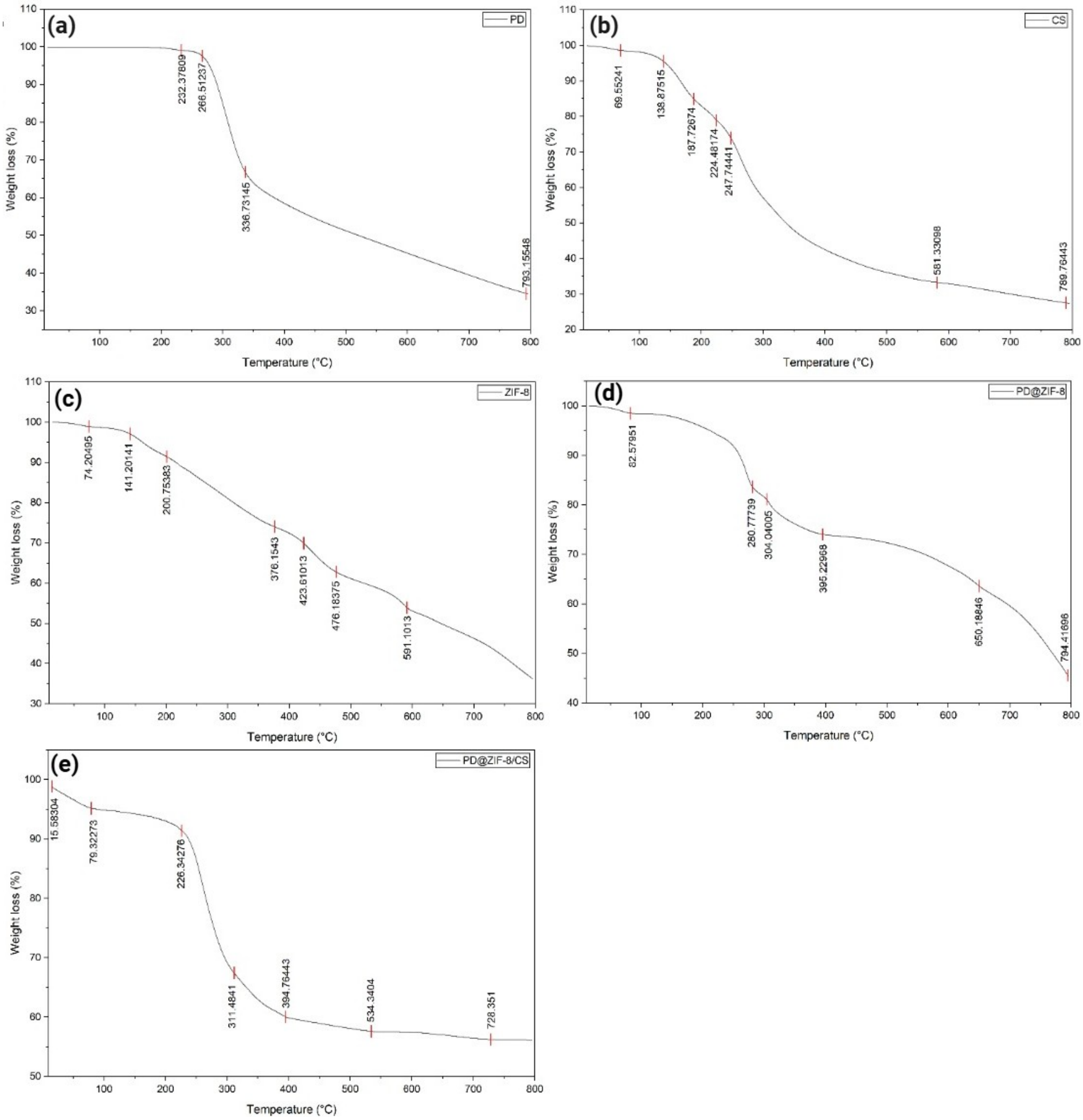

Figure S6 Thermogravimetric analysis (TGA) curves of pure PD, CS, ZIF-8, PD@ZIF-8, and PD@ZIF-8/CS composites recorded under a nitrogen atmosphere. PD exhibits a major decomposition between 266–336 °C corresponding to glycosidic bond cleavage and stilbene oxidation, while CS shows a multistep degradation typical of polysaccharides. ZIF-8 displays high thermal stability with framework decomposition above 300 °C due to linker breakdown. Encapsulation of PD into ZIF-8 (PD@ZIF-8) reduces the overall mass loss and shifts decomposition to higher temperatures, confirming confinement-induced stabilization. The chitosan-coated hybrid (PD@ZIF-8/CS) demonstrates the highest thermal resistance and maximum char residue, indicating synergistic barrier and char-forming effects of CS on the ZIF-8 matrix.

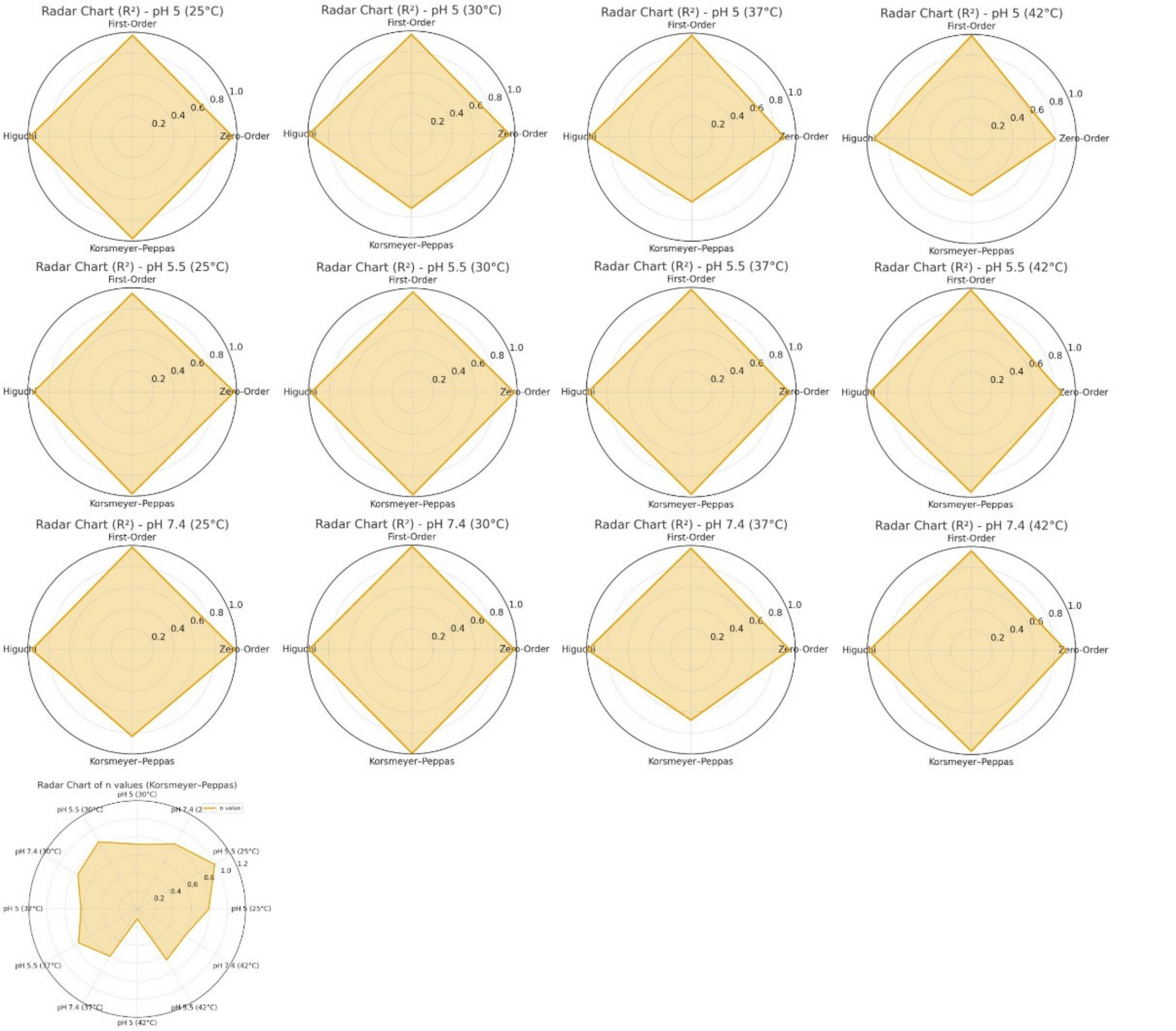

Figure S7 Radar charts of drug release kinetics from PD@ZIF-8: Illustrating the correlation coefficients (R^2) of different drug release kinetic models, Zero-order, First-order, Higuchi, and Korsmeyer–Peppas for ZIF-8 at varying pH conditions (5.0, 5.5, and 7.4) and temperatures (25 °C, 30 °C, 37 °C, and 42 °C). The shaded regions represent the relative fitting performance of each model, indicating the most appropriate kinetic behavior under specific experimental conditions. The lower radar chart depicts the diffusion exponent (n) values obtained from the Korsmeyer–Peppas model, providing further insight into the drug release mechanism and its dependence on pH and temperature.

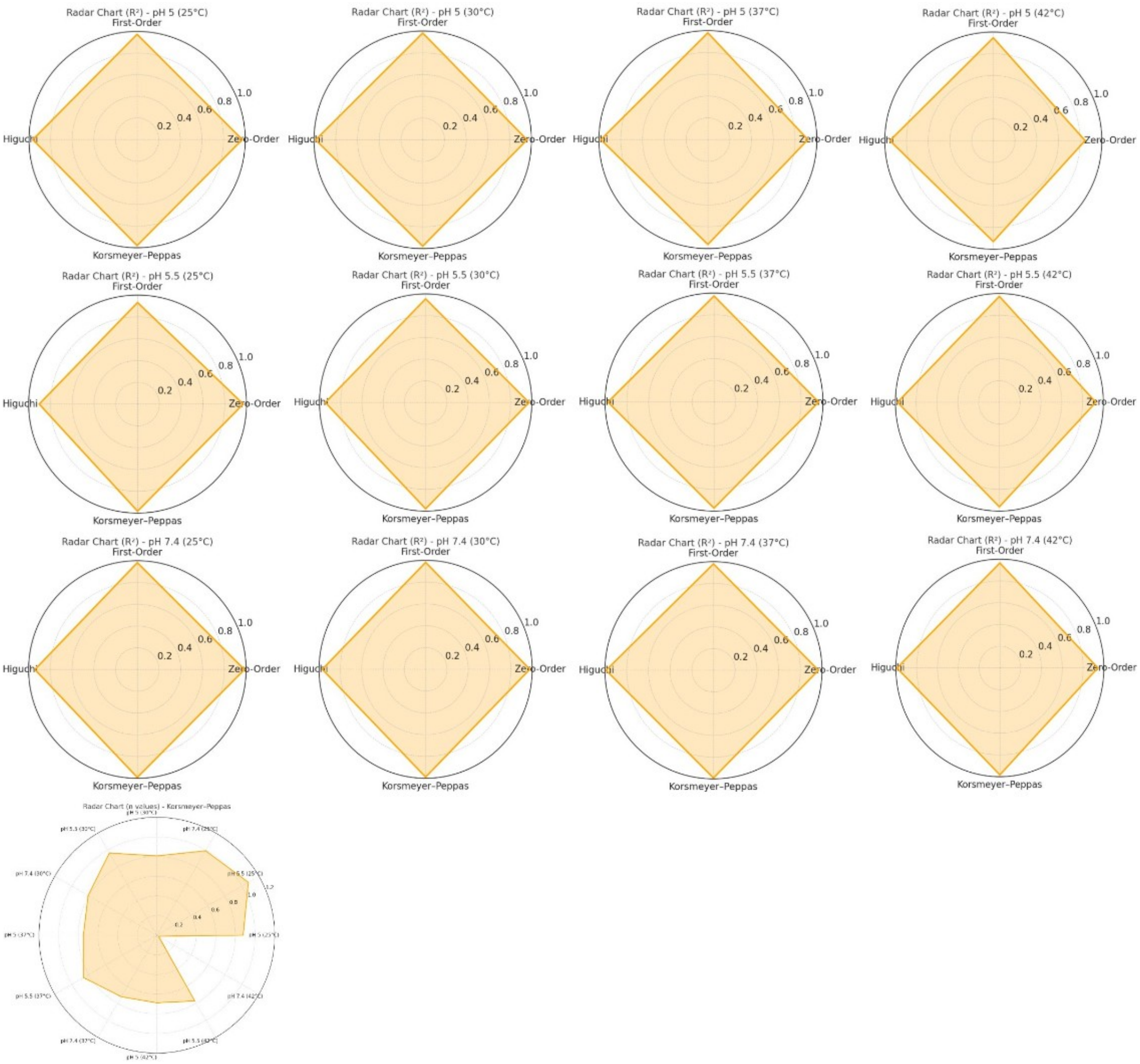

Figure S8 Radar charts of drug release kinetics from PD@ZIF-8/CS: Radar charts showing the correlation coefficients (R^2) of various kinetic models, Zero-order, First-order, Higuchi, and Korsmeyer–Peppas for PD@ZIF-8/CS under different pH (5.0, 5.5, and 7.4) and temperature (25 °C, 30 °C, 37 °C, and 42 °C) conditions. The shaded regions indicate the comparative fitting efficiency of each kinetic model, identifying the most suitable model under specific release environments. The lower radar chart summarizes the diffusion exponent (n) values derived from the Korsmeyer–Peppas model, providing insights into the release mechanism and its modulation by pH and temperature for the PD@ZIF-8/CS composite.
